# Sulforaphane-Induced Klf9/Prdx6 Axis Acts as a Molecular Switch to Control Redox Signaling and Determines Fate of Cells

**DOI:** 10.3390/cells8101159

**Published:** 2019-09-27

**Authors:** Bhavana Chhunchha, Eri Kubo, Dhirendra P. Singh

**Affiliations:** 1 Department of Ophthalmology and Visual Sciences, University of Nebraska Medical Center, Omaha, NE 68198, USA; bchhunchha@unmc.edu; 2 Department of Ophthalmology, Kanazawa Medical University, Ishikawa 9200293, Japan; kuboe@kanazawa-med.ac.jp

**Keywords:** oxidative stress, sulforaphane, Prdx6, Nrf2, Klf9, antioxidants

## Abstract

Sulforaphane (SFN), an activator of transcription factor Nrf2 (NFE2-related factor), modulates antioxidant defense by Nrf2-mediated regulation of antioxidant genes like *Peroxiredoxin 6* (*Prdx6*) and affects cellular homeostasis. We previously observed that dose levels of SFN are crucial in determining life or death of lens epithelial cells (LECs). Herein, we demonstrated that higher doses of SFN (>6 μM) activated death signaling by overstimulation of Nrf2/ARE (antioxidant response element)-mediated Kruppel-like factor (Klf9) repression of *Prdx6* expression, which increased reactive oxygen species (ROS) load and cell death. Mechanistically, Klf9 bound to its repressive Klf9 binding elements (RKBE; 5-C^A/G^CCC-3) in the *Prdx6* promoter, and repressed Prdx6 transcription. Under the condition of higher dose of SFN, excessive Nrf2 abundance caused death signaling by enforcing Klf9 activation through ARE (5-RTGAYnnnGC-3) in Klf9 promoter that suppress antioxidant genes such as *Prdx6* via a Klf9-dependent fashion. Klf9-depletion showed that Klf9 independently caused ROS reduction and subsequent cell survival, demonstrating that Klf9 upregulation caused cell death. Our work revealed the molecular mechanism of dose-dependent altered activity of SFN in LECs, and demonstrated that SFN activity was linked to levels of Nrf2/Klf9/Prdx6 axis. We proposed that in the development of therapeutic interventions for aging/oxidative disorders, combinations of Klf9-ShRNA and Nrf2 inducers may prove to be a promising strategy.

## 1. Introduction

Substantial experimental evidence indicates that etiopathobiology of age-related disorders is associated with increased levels of reactive oxygen species (ROS) and reduced expression and activity of antioxidant pathway, leading to molecular damage that result in pathobiology and disease. To cope with internal and external oxidative stresses, cells have evolved defensive mechanisms that integrate the coordinated stimulation of a group of phase I and phase II antioxidant enzymes, such as NAD(p)H quinone oxidoreductase (NQO1), Thioredoxin reductases (Txnrds) Glutathione peroxidase (GPx), Catalase (Cat), Superoxide dismutases (SODs) and peroxiredoxin 6 (Prdx6). These enzymes are upregulated by Nrf2 (NFE2-related factor 2), a universal transregulator of antioxidant response genes, to revive cellular redox homeostasis [1,2,3,4]. The Nrf2-mediated protective pathway is highly sensitive to oxidative stress in cells [5]. To maintain homeostasis, Nrf2 binds to antioxidant response elements (ARE) present in antioxidant protein genes and upregulates their transcription. Dysregulation of Nrf2 during aging or oxidative stress results in failure of antioxidant response-mediated cellular homeostasis [1].

Recent evidence reveals that oxidative stress is a major culprit in aging disorders, and that the ability to prevent or blunt oxidative stress is a key determinant of aging diseases; thus, strategies should be aimed at limiting oxidative load or enhancing the cellular host defense system. Boosting or reactivating the defense system by means of Nrf2-mediated antioxidant pathway appears to be a reasonable and practical approach to reducing oxidative-induced injurious signaling. Work by our lab and others has shown that naturally occurring compounds such as sulforaphane (SFN), curcumin and α-lipoic acid have the ability to reactivate Nrf2/ARE-mediated antioxidant pathway and provide cytoprotection against pathobiology and cell damage induced by aging and oxidative stress [1,6,7,8,9,10,11,12,13]. Using lens epithelial cells (LECs) and SFN as a model system, we found that lower concentrations of SFN provided increased cell growth and cytoprotection by upregulating the Nrf2/ARE antioxidant pathway, but higher concentrations had the opposite effect [1]. This bimodal phenomenon of SFN has been well-documented [14,15,16,17,18,19]. However, recently, we observed that at higher concentrations, SFN augmented the expression of *Nrf2*, but surprisingly caused a dramatic reduction in expression of its target antioxidant gene *Prdx6*, such as Prdx6 resulting in cell death. The Nrf2/ARE pathway has a cytoprotective role in maintaining redox homeostasis [20,21,22,23,24]. Nonetheless, upregulation of Nrf2 (beyond physiological levels) has been shown to be ineffective in reducing oxidative load and improving cell health [25]. These studies provided the basis of the current investigation of the molecular mechanism underlying in excessive activation of Nrf2-mediated dysregulation of Prdx6 in hLECs (human lens epithelial cells).

Nrf2 is a major sensor of oxidative stress in the cells and a master regulator of antioxidant defense pathway [2,26,27,28]. Bioavailable levels of Nrf2 are regulated through cytosolic binding to Kelch-like-ECH-associated protein-1 (Keap1) by preventing its proteosomal degradation under normal/basal physiological condition [29,30]. With mild to moderate or high levels of oxidative stress or with use of Nrf2 inducers, Nrf2-Keap1 interaction is disrupted and Nrf2 is accumulated in nucleus in a dose-dependent manner [1]. However, under excessive oxidative stress, the excessive nuclear Nrf2 binds to the regulatory region of Kruppel-like factor 9 (*Klf9*) gene promoter and transactivates its expression, which suppresses some of the antioxidant genes by binding to their repressive sites (5′-C^A/G^CCC-3′) [26,31] and causes cellular injury [32].

A ubiquitously expressed member of an evolutionarily conserved transcriptional regulator KLF family, Klf9 is also designated as BTEB1 (basic transcription element binding protein 1). Klf9 can act as either a repressor or an activator of gene transcription. This activity of Klf9 is related to the number of binding sites, GC-boxes, present in gene promoter and cell types [33]. Studies have shown that Klf9 activates promoter activity with repeated GC boxes, while promoters with only one GC box can be repressed by Klf9 [33]. Klf9 acts by modulating gene transcription, and in doing so, plays a role in cell growth and cell death, in cell development and differentiation and in cancer [34,35,36,37,38]. Klf9 represses multiple anti-oxidant defense genes including mitochondrial thioredoxin reductase2 (TXNRD2), leading to increased ROS levels [26,39,40]. Conserved Nrf2/ARE interaction sites have been demonstrated in the regulatory region of *Klf9* promoter [13,41,42]. Based on the existing research, we postulated that the antioxidant *Prdx6* gene promoter might bear Klf9 binding elements. In silico analysis of a *Prdx6* promoter revealed that, indeed, the *Prdx6* promoter has several Klf9 binding sites of single GC boxes (but not tandem repeats), suggesting that Klf9 may act as repressor for *Prdx6* gene transcription in excessive oxidative load (current study).

The multitasking protective protein Prdx6, with its glutathione (GSH) peroxidase and acidic Ca^2+^-independent phospholipase A_2_ activities, belongs to the Prdx family. The six members of the Prdx family are classified based on the number of cysteine (Cys) residues. Prdx6 contains 1-Cys at position 47 (Cys47), while Prdx1-5 have 2-Cys residues. Prdx6 is a cytosolic protein and is abundantly expressed in lung, eye lens, keratinocytes, skin and brain [43,44,45,46]. It is also localized in ROS-producing organelles, such as endoplasmic reticulum, plasma membrane, lysosomes, mitochondria and cerebrospinal fluid [47,48,49,50,51], suggesting its importance in controlling redox-homeostasis for cell survival [1,48,52,53,54]. Prdx6 protects many cell types from lipid peroxidation-mediated damage to membrane, DNA and protein [1,6,7,49,52,55,56,57,58]. *Prdx6* is transcriptionally regulated by Nrf2, and dysregulation of Nrf2 during aging causes reduction in *Prdx6* expression that leads to increased ROS-induced cell death [1]. Loss of Prdx6 leads to cell death, tissue degeneration and development of many types of disorders, including oxidative-induced cataract [43,59,60], psoriasis [61] and atherosclerosis [62].

SFN is a naturally occurring organosulfur found in cruciferous vegetables, with high levels detected in broccoli and broccoli sprouts. Recent evidence shows SFN′s diversified activities, ranging from cell survival and cytoprotection to cytotoxicity and growth inhibition; these activities are determined by concentrations of SFN and cellular background [1,63,64,65,66,67]. SFN exerts its function via activating the redox-sensitive Nrf2/ARE antioxidant pathway and interacting with other mammalian biochemical pathways [63,68,69]. Activation of Nrf2 by SFN occurs through increased cytosolic transcription and facilitation of Nrf2 translocation in nucleus by inducing cysteine modifications to Keap1 [30,70]. A greater understanding of how SFN produces its bimodal effects and directs survival or death signaling pathways in LECs or redox active cells is needed. In the research reported here, we used human LECs as a model system to reveal the dose-dependent molecular mechanism of SFN in regulation of Nrf2/ARE-mediated protective pathway. We found that the cytotoxic effect of SFN at high doses was linked to repression of *Prdx6* and dramatic increase in ROS levels with aberrant expression and nuclear accumulation of *Nrf2*. This accumulation of *Nrf2* led to its binding to ARE present in the promoter of unfavorable target *Klf9* and enhanced its expression and nuclear abundance. We found that Klf9 bound to its Repressive Klf9 Binding Elements (RKBE) of the *Prdx6* promoter and repressed the *Prdx6* expression that in turn resulted in increased ROS-induced cell injury, suggesting an advent of dominant Klf9-mediated repressive signaling during high doses of SFN. Klf9 depletion experiments showed that Klf9 dramatically reduced ROS levels and cell injury. Aberrant *Klf9* expression induced by higher doses of SFN was a major culprit in acceleration of ROS generation and cell death, while lower doses of SFN did not affect *Klf9* expression, and led to SFN-induced Nrf2/ARE-mediated cellular protection.

## 2. Materials and Methods

### 2.1. Cell Culture 

hLECs were derived from 12 infants who underwent surgery for retinopathy of prematurity [71] (a kind gift of Dr. Venkat N. Reddy, Eye Research Institute, Oakland University, Rochester, MI, USA). These LECs were immortalized with SV40 and were maintained in Dulbecco′s Modified Eagle Medium (DMEM; Invitrogen, Waltham, MA, USA) with 15% fetal bovine serum (FBS; Atlanta Biologicals, Atlanta, GA, USA), 100 µg/mL streptomycin, and 100 µg/mL penicillin in 5% CO_2_ environment at 37 °C as described previously [7,57]. To examine the effect of Sulforaphane (SFN), cells were treated with different concentrations (DMSO (dimethyl sulfoxide; Sigma Aldrich, St. Louis, MO, USA), 6, 12, 18 and 24 µM in complete medium) of SFN. A stock solution of SFN (50 mM) was prepared in DMSO and diluted in culture medium keeping the final DMSO concentration at < 0.05% and same concentration of DMSO was used as vehicle control. DL-SFN (Catalog no. S4441) and tBOOH (tert Butyl hydroperoxide; Catalog no. 458139) were purchased from Sigma Aldrich (St. Louis, MO, USA).

### 2.2. Quantitation of Intracellular ROS Level by H2-DCF-DA and CellROX^®^ Deep Red Reagent

Intracellular ROS level was measured by use of fluorescent dye dichlorofluorescin diacetate (H2DCFDA), a nonpolar compound that is converted into a polar derivative (dichlorofluorescein) by cellular esterase after incorporation into cells [7,52,72,73]. On the day of the experiment, the medium was replaced with Hank′s solution containing 10 µM H2DCFDA dye and cells were incubated at 37 °C. After 30 min, intracellular fluorescence was detected with excitation (Ex) at 485 nm and emission (Em) at 530 nm by a Spectra Max Gemini EM (Mol. Devices, Sunnyvale, CA, USA).

ROS level were measured according to the company′s protocol (CellROX^®^ Deep Red Oxidative Stress Reagent, Catalog No. C10422) and described in previous protocol [74]. In brief, LV Sh-control and LV Sh-Klf9 hLECs (5 × 10^3^) cells were seeded in 96 well plate and 24 h later treated with different concentrations of SFN as indicated. After 48 h, CellROX deep red reagent was added with a final concentration of 5 µM and cells were incubated at 37 °C for 30 min. Media containing CellROX deep red reagent were removed and fixed with 3.7% formaldehyde. After 15 min, fluorescence signal were measured at Ex 640 nm/ Em 665 nm with Spectra Max Gemini EM (Mol. Devices, Sunnyvale, CA).

### 2.3. Cell Survival Assay [(3-(4,5-Dimethylthiazol-2-yl)-5-(3-carboxymethoxyphenyl)-2 to 4-sulphophenyl) 2H-tetrazolium Salt (MTS) Assay] 

A colorimetric MTS assay (Promega, Madison, WI, USA) was performed as described earlier [7,75,76]. This assay of cellular viability uses MTS and an electron coupling reagent (Phenazine ethosulfate; PES). PES has enhanced chemical stability, which allows it to be combined with MTS to form a stable solution. Assay are performed by adding MTS reagent directly to culture cells, incubating for 1–4 h and then recording absorbance at 490 nm with a 96-well plate reader, Spectra Max Gemini EM (Mol. Devices, Sunnyvale, CA). Results were normalized with absorbance of the untreated control(s).

### 2.4. Real-Time Quantitative Reverse Transcriptase-Polymerase Chain Reaction (RT-qPCR)

Total RNA from the cultured hLECs untreated and/or treated with SFN (DMSO, 6, 12, 18 and 24 µM) were isolated using the single-step guanidine thiocyanate/phenol/chloroform extraction method (Trizol Reagent, Invitrogen, Waltham, MA, USA). To examine the levels of Nrf2, Klf9 and Prdx6, 0.5 to 5 micrograms of total RNA was converted to cDNA using Superscript II RNAase H-reverse-transcriptase. Real-time quantitative PCR was performed with SYBR Green Master Mix (Roche Diagnostic Corporation, Indianapolis, IN, USA) in a Roche^®^ LC480 Sequence detector system (Roche Diagnostic Corporation). PCR conditions of 10 min (min) hot start at 95 °C, followed by 45 cycles of 10 s (sec) at 95 °C, 30 sec at 60 °C and 10 sec at 72 °C. The primer Sequence was: Nuclear factor (erythroid-derived 2)-like 2 (Nrf2), Forward Primer: 5′-TGC TTT ATA GCG TGC AAA CCT CGC-3′; Reverse Primer: 5′-ATC CAT GTC CCT TGA CAG CAC AGA-3′; Klf9, Forward Primer: 5′-CTGGTTGCTGGGACTGTAGC-3′; Reverse Primer: 5′-GTTTTCCAGCTCCCAAACAG-3′; Prdx6, Forward Primer: 5′-GCATCCGTTTCCACGACT-3′ and Reverse Primer: 5′-TGCACACTGGGGTAAAGTCC-3′; β-actin, Forward Primer: 5′-CCAACCGCGAGAAGATGA-3′ and Reverse Primer: 5′-CCAGAGGCGTACAGGGATAG-3′.

The relative quantity of the mRNA was obtained using the comparative threshold cycle (CT) method. The expression levels of target genes were normalized to the levels of β-actin as an endogenous control in each group.

### 2.5. Extraction of Nuclear and Cytosolic Fraction and Total Cell Lysates

Nuclear extract was prepared following as described earlier [52,77]. Briefly, LECs (1 × 10^6^) were cultured in 100-mm plates. hLECs were treated with DMSO control or different concentrations of SFN (6, 12, 18 and 24 µM) for 24 h. The cells were washed gently with chilled phosphate-buffered saline (pH 7.4). Cells were collected by centrifugation using a micro-centrifuge and resuspended in 5 pellet volumes of cytoplasmic extract buffer ((10 mM 4-(2-hydroxyethyl)-1-piperazineethanesulfonic acid (HEPES, adjusted pH at 7.9), 10 mM KCl, 0.1 mM EDTA (Ethylenediaminetetraacetic acid), 0.4% (v/v) Nonidet P-40, 0.5 mM phenylmethylsulfonyl fluoride (PMSF), 1 mM DTT (Dithiothreitol) and Protease inhibitor). After a short incubation on ice and centrifugation (4 °C) at 10000 rpm for 10 min, the cytoplasmic extract was transfer in fresh tube from the pellet. Following careful washing with cytoplasmic extract without detergent (Nonidet P-40), the fragile nuclei were resuspended in nuclear extract buffer ((20 mM HEPES (adjusted pH at 7.9), 0.4 M NaCl, 1 mM EDTA, 10% (v/v) glycerol, 1 mM DTT, 0.5 mM PMSF and Protease Inhibitor) and incubated for 2 h at 4 °C with continuous vortexing. Finally, the extract was spun at 14,000 rpm for 15 min to pellet the nuclei. After centrifugation, the nuclear extract was transferred and aliquoted in fresh tubes, and individual aliquots were stored at −70 °C to avoid repeated freezing and thawing of the preparation.

Total cell lysates of LECs were prepared in ice-cold radioimmune precipitation buffer (RIPA buffer) [58]. Briefly, LECs (1 × 10^6^) were cultured in 100-mm plates. The cells were washed gently with chilled phosphate-buffered saline (pH 7.4). Cells were collected by centrifugation using a micro-centrifuge and resuspended in three pellet volumes of RIPA buffer *(1% IGEPAL (CA-630; Sigma Aldrich, St. Louis, MO, USA) 0.5% sodium deoxycholate, 0.1% sodium dodecyl sulfate (SDS; Catalog No. 15553027, Thermo Fisher Scientific, Waltham, MA, USA), 0.5 mM phenylmethylsulfonyl fluoride (PMSF), and Protease inhibitor). After 30 min of incubation on ice and centrifugation (4 °C) at 10,000 rpm for 10 min, the total cell lysates was transfer in fresh tube from the pellet. Protein level was estimated according to the Bradford protein assay and/or Pierce™ BCA Protein assay methods and extract was used for experiment as required.

### 2.6. Protein Expression Analysis

Total cell lysates, cytosol and nuclear extract of LECs were prepared and protein blot analysis was performed as described previously [43,52,78,79]. The membranes were probed with anti-Nrf2 (SC-722, Santa Cruz Biotechnology, Dallas, TX, USA), Anti-Klf9 (ab177158, Abcam^®^, Cambridge, MA, USA), Anti-Prdx6 antibody (LF-PA0011, Ab Frontier, South Korea) or β-actin (A2066, Sigma-Aldrich, St. Loius, MO, USA)/Lamin B1 (ab133741, Abcam^®^, Cambridge, MA, USA) as internal control to monitor those protein expressions. After secondary antibody (sc-2354 and sc-2768, Santa Cruz Biotechnology, Dallas, TX, USA), protein bands were visualized by incubating the membrane with luminol reagent (sc-2048; Santa Cruz Biotechnology, Dallas, TX, USA) and images were recorded with a FUJIFILM-LAS-4000 luminescent image analyzer (FUJIFILM Medical Systems Inc., Hanover Park, IL, USA).

### 2.7. Transcription Factor Nrf2 Activation Assay

Nrf2 activation assay was performed according to manufacturer′s protocol (TransAM Nrf2 Transcription Factor Assay Kit, Cat No 50296, Active motif, Carlsland, California, USA). In brief, 10 µg of nuclear extract (up to 10 µL diluted with complete lysis buffer) prepared from SFN treated hLECs added to the strips well, following the addition of 40 µL complete binding buffer contains 20 pmol of the wild-type and/or mutated consensus oligonucleotide to each sample well. For blank well, 10 µL of complete lysis buffer were used. The plate was incubated for 1 h at room temperature (RT) with mild agitation. 100 µL primary antibody (1:1000 in 1× antibody binding buffer) added after three washes with 1× washing buffer and incubated at RT for 1 h without agitation. 100 µL of diluted anti-rabbit HRP (horseradish peroxidase)- conjugated antibody (1:1000 dilution in 1× antibody binding buffer) after three washing was added and incubated for 1 h at RT. 100 µL of developing solution was added to wells after four washing and incubated at RT in dark for 2 to 10 min. Finally, by addition of 100 µL of stop solution, optical density (O.D.) was recorded at absorbance 450 nm.

### 2.8. Chromatin Immunoprecipitation (ChIP) Assay

ChIP was performed using the ChIP-IT^®^ Express (Cat. No. 53008; Active Motif, Carlsbad, CA, USA) and ChIP-IT^®^ qPCR analysis kit (Cat. No. 53029; Active Motif, Carlsbad, CA, USA) following the manufacturer′s protocol and as described earlier [1,7]. The following antibodies were used: control IgG and antibody specific to Klf9 (Catalog No. 701888, Thermo Scientific, Waltham, MA, USA) and Nrf2 (Catalog No. Ab180845, Abcam). RT-PCR amplification was carried out using 5 μL of DNA sample with primers as indicated below. The program for quantification amplification was 3 min 94 °C, 20 sec at 95 °C, 30 sec at 59 °C and 30 sec at 72 °C for 36 cycles in 25 μL reaction volume (RT-PCR). Data obtained with RT-PCR run on 1% agarose gel and visualized band under UV and image captured. For qPCR 2 min at 95 °C, 15 sec at 95 °C, 20 sec at 58 °C and 20 sec at 72 °C for 40 cycles in 20 μL reaction volume (qPCR). Data obtained from qPCR presented as histogram. Primers as below,

Klf9 ARE3 (ChIP-Primer):

Forward: 5′-CGCTAGAGTTACGAAACAGGG-3′; Reverse: 5′-GAAAGGCCATCCGTTCATGC-3′

Klf9 ARE4 (ChIP-Primer):

Forward: 5′-GCGCCAGCACCCGGCCGAACC-3′; Reverse: 5′-GCTGGTGTTGCTGTCCCTGG-3′

Prdx6 RKBE1 (ChIP-Primer):

Forward: 5′-GCTGTGCGAAGCCGCCGCA-3′; Reverse: 5′-GAAGCTTGAGGATGCGCCA-3

Prdx6 RKBE2 (ChIP-Primer):

Forward: 5′-GTAGTCGAGCAGTCACTCCA-3′; Reverse: 5′-GAAGGAAGAGGAACGCGGCAG-3

Prdx6 RKBE3 (ChIP-Primer):

Forward: 5′-GGTTCATAACAAACAGAAAGG-3′; Reverse: 5′-AGCCCAGCTACGATGAACTG-3

Prdx6 RKBE4 (ChIP-Primer):

Forward: 5′-GTCTGTCACCGGTTTCCCTT-3′; Reverse: 5′-GAGACCTACTGTGTGCAGGT-3

Prdx6 RKBE5 (ChIP-Primer):

Forward: 5′-CAGAGCACCTACCGTGAGCT-3′; Reverse: 5′-GAAGGGAAACCGGTGACAGA-3

### 2.9. Construction of Mouse Klf9 Promoter-Chloramphenicol Acetyltransferase (CAT) Reporter Vector

5′-Flanking region of mouse *Klf9* gene ranging from −5856 to +71bp was isolated from mouse genomic DNA by using an Advantage^®^ Genomic PCR Kit (Cat. No. 639103 & 639104, Clontech Laboratories, Inc, Mountain View, CA, USA, 94043). A construct of −5856 to +71 bp was constructed by ligating this DNA fragment into CAT reporter plasmid (p), basic pCAT vector (Promega) using *MluI* and *XhoI* sites. The plasmid was amplified and sequenced. Primers used for isolating the genomic DNA fragment were as follows: Forward Primer (*MluI* site): 5′-AAAA*ACGCGT*GGTCATCGTAGGAAAGATGTGG-3′ and Reverse Primer (*XhoI* site) 1: 5′-AAAA*CTCGAG*CCTACGAGACACTTCTTCCC-3′.

### 2.10. Construction of Human Prdx6 Promoter-Chloramphenicol Acetyltransferase (CAT) Reporter Vector

The 5′-flanking region spanning from −1559 to +30 bp was isolated from human genomic DNA by using an Advantage^®^ Genomic PCR Kit (Cat. No. 639103 & 639104, Clontech Laboratories, Inc, Mountain View, CA 94043). PCR product was cleaned and verified by sequencing as described previously [80]. A construct containing −1559 to +30 bp was engineered by ligating it to basic pCAT vector (Promega) using the *SacI* and *XhoI* sites. Primers were as follows: Sense; 5′-GACAGAGTTGAGCTCCACACAG-3′; and antisense; 5′-CACGTCCTCGAGAAGCAGAC-3′.

### 2.11. Site-Directed Mutagenesis (SDM)

PCR-based site-directed mutagenesis was carried out using the QuikChange^™^ lightning site-directed mutagenesis kit (Agilent Technologies; Catalog No. 210518, Santa Clara, CA, USA), following the company′s protocol. Briefly, amino acid exchanges at the Klf9 sites (RKBE 1 and 3) mutants (RKBE 1; GC to TT and RKBE 3; AC to TT) were generated by point mutations in the human promoter of Prdx6-CAT plasmid. Nrf2/ARE (3 and 4) sites mutants (T to G) generated by point mutation in the mouse *Klf9* promoter of Klf9-CAT reporter plasmid. The following complementary primers were used (changed nucleotides are in red boldface type and underlined):

Klf9 ARE site3 mutant (ARE3-mut; T to G, -5213 to -5203):

Forward primer: 5′-CTGTCCTCAAAGGAACCTGCCTCCTC-3′

Reverse primer: 5′-GAGGAGGCAGGTTCCTTTGAGGACAG-3′

Klf9 ARE site4 mutant (ARE4-mut; A to C, -5808 to -5798):

Forward primer: 5′-CGATTCCTGCAAAGTCCTCTCCACTCGCAC-3′

Reverse primer: 5′-GTGCGAGTGGAGAGGACTTTGCAGGAATCG-3′

Prdx6 RKBE1 mutant (RKBE1-mut; GC to TT, -407 to -403): 

Forward primer: 5′-CCCTAAAGCGCGTACTTCCTGCAGAGTCAAACC-3′

Reverse primer: 5′-GGTTTGACTCTGCAGGAAGTACGCGCTTTAGGG-3

Prdx6 RKBE3 mutant (RKBE3-mut; AC to TT, -700 to -696):

Forward primer: 5′-CTCTGACATAAGGTCTTCCATACTTCTGGGTC-3′

Reverse primer: 5′-GACCCAGAAGTATGGAAGACCTTATGTCAGAG-3

### 2.12. Lentiviral (LV) Infection

CopGFP control lentiviral particle (LV Sh-Control, Sc-108084) and BTEB1 (Klf9)/GFP ShRNA (LV Sh-Klf9, sc-37716-VS) were purchased from Santa Cruz Biotechnology and hLECs were infected following the Company′s protocol. Briefly, hLECs were cultured in 12 well plate in complete medium. After 24 h, media were removed and then substituted with 1 mL medium containing polybrene (sc-134220, Santa Cruz) at a final concentration of 5 µg/mL. Cells were infected by adding the Sh-Control and Sh-Klf9 lentiviral particles to the culture, mixed by swirling and incubated overnight. At 24 h post infection, polybrene-containing medium were removed and fresh complete medium (without polybrene) were added. Infected cells were split and incubated in complete medium for a further 24–48 h. For stable selection, the infectants were treated with puromycin dihydrochloride (sc-108071) selection marker. These stably infected hLECs with LV Sh-Control or LV Sh-Klf9 were used for the current studies.

### 2.13. Statistical Analysis

For all quantitative data collected, statistical analysis was conducted by Student′s t test and /or one-way ANOVA when appropriate, and was presented as mean ± S.D. of the indicated number of experiments. A significant difference between control and treatment group was defined as a *p* value of <0.05 and 0.001 for two or more independent experiments.

## 3. Results

### 3.1. High Doses of SFN Increased Generation of Intracellular ROS and Were Linked to LEC Death

**Figure 1:**
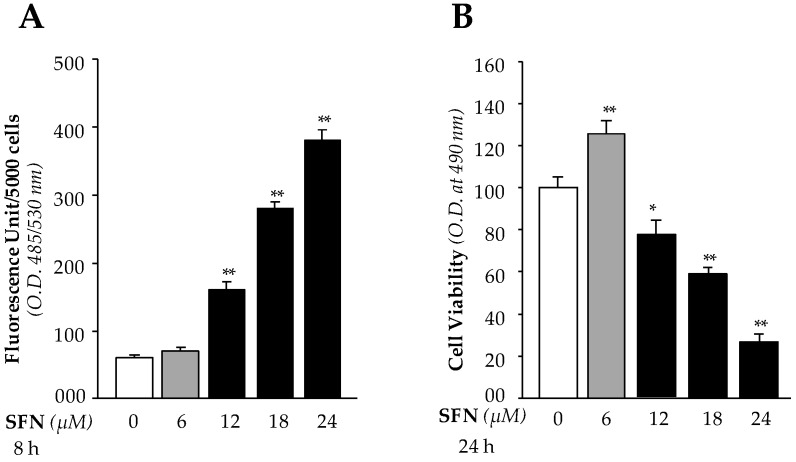
Lethal doses of SFN increased accumulation of ROS and reduced cell viability. Cultured hLECs were treated with increasing amounts of SFN as indicated. (**A**) After 8 h, ROS were quantified by H2-DCF-DA dye as described in “Materials and Methods” section. (**B**) MTS assay was conducted to monitor cell viability against oxidative stress after 24 h. The data represent the mean ± S.D. from three independent experiments. * *p* < 0.05, ** *p* < 0.001; SFN treated versus untreated (DMSO) control.

We recently showed [1] that SFN activates Nrf2/ARE antioxidant pathway of LECs. During this study we observed increased LECs death with increased SFN concentrations (> 6μM). Because SFN is known to protect cells against oxidative stress through upregulation of the Nrf2/ARE antioxidant pathway, we sought to determine the mechanism by which high doses of SFN regulate ROS levels in LECs. We first identified an effective noncytotoxic dose of SFN, finding that doses of less than 6 µM were effective noncytotoxic concentrations, while doses of more than 6 µM inhibited LEC growth and increased cell death. We hypothesized that higher doses of SFN may induce increased levels of ROS that in turn lead to death of LECs. We treated hLECs with different concentrations of SFN (6 µM non-cytotoxic and 12 µM, 18 µM and 24 µM cytotoxic) and measured ROS and cell viability using H2-DCF-DA dye and MTS assays, respectively. With higher concentrations, we observed dose-dependent significant increases in accumulation of ROS (Figure 1A) and cell death (Figure 1B). In contrast, noncytotoxic concentrations of SFN did not alter ROS (Figure 1A), and increased cell growth (Figure 1B). These observations served as a basis for study of the molecular mechanism of SFN-mediated bimodal function.

### 3.2. High Doses of SFN Induced a Dramatic Rise in Nrf2/Klf9 Expression with Reduced Prdx6 Expression and Increased Accumulation of Nrf2/Klf9 in the Nucleus

**Figure 2:**
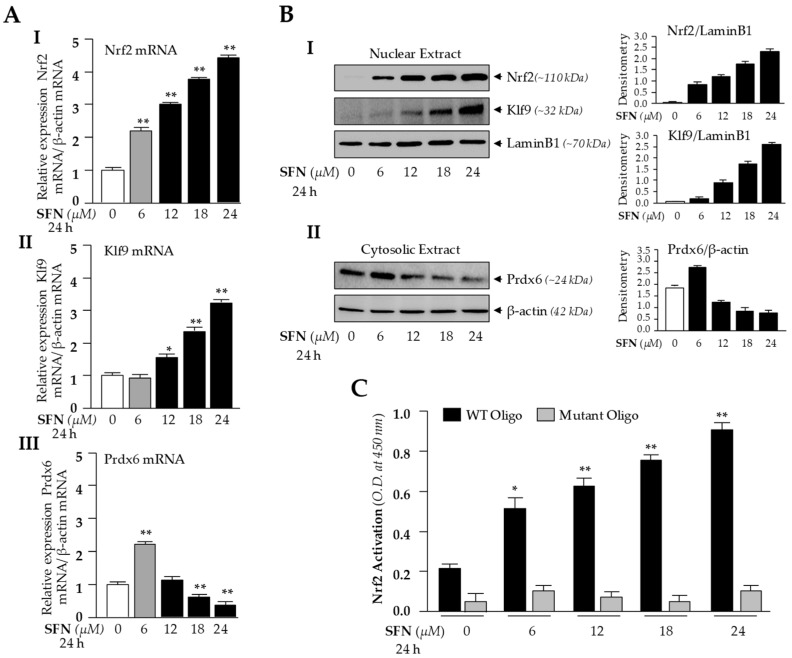
High dose of SFN increased expression of *Nrf2* and *Klf9* and reduced *Prdx6* expression. (**A**) Cells were untreated or treated with increasing doses of SFN; non-toxic amount of SFN (<6 μM) and higher doses of SFN (>12 μM) as indicated. After 24 h, RT-qPCR was conducted using primers specific to *Nrf2* (AI), *Klf9* (AII) and *Prdx6* (AIII). Experiments were conducted three times and data is represented as mean ± S.D. * *p* < 0.05, ** *p* < 0.001; SFN treated versus untreated (DMSO) control. (**B**) Nrf2 and Klf9 are overly accumulated in nucleus and inversely related to reduce abundance of Prdx6 protein in response to excessive amount of SFN. hLECs were treated or untreated with different doses of SFN as indicated. Nuclear (Nrf2 and Klf9) and cytosolic (for Prdx6) fractions were isolated. Samples containing equal amounts of protein were immunoblotted using an antibody specific to Nrf2, Klf9 and Prdx6, as shown. A dramatic nuclear accumulation of Nrf2 and Klf9 (BI) and reduction in *Prdx6* expression (BII) were observed in response to increased concentrations of SFN treatment. Right adjacent panel reveals densitometry analysis of each protein bands. (**C**) SFN treated hLECs displayed significant increased Nrf2 activity. hLECs were treated with DMSO control or different concentration of SFN for 24 h, and nuclear extracts were analyzed for Nrf2-ARE (antioxidant responsive element) binding by ELISA (enzyme-linked immunosorbent assay). Nuclear extracts containing equal amounts of protein were processed and assayed for Nrf2 activity using a commercially available kit (Active motif) as described in Materials and Methods. The data represent the mean ± S.D. from three independent experiments. *p* values were determined SFN treated versus untreated (DMSO) control. * *p* < 0.05, ** *p* < 0.001.

Based on these findings (Figure 1), we surmised that the Nrf2/ARE antioxidant pathway in LECs may have been suppressed, as previously reported in other cell types [26]. To determine the molecular mechanism by which ROS was increased (Figure 1A), we examined the dose-dependent effect of SFN on Nrf2/Klf9 mRNA and protein levels by using the same concentrations shown in Figure 1. We treated hLECs for 24 h with increasing amounts of Nrf2 activator SFN [1,81], as shown in Figure 2, and conducted RT-qPCR. Levels of Nrf2 and its target gene, *Prdx6* mRNA expression, were increased in response to 6 μM of SFN treatment, but Klf9 levels were not upregulated (Figure 2A, gray bar). Treatment with doses higher than 6 μM of SFN resulted in dose-dependent Klf9 upregulation, which was correlated with increased *Nrf2* expression and reduced *Prdx6* expression (Figure 2A, black bars).

To examine intracellular fate and status of nuclear Nrf2, Klf9 and cytoplasmic Prdx6 protein in hLECs in response to SFN treatment, we analyzed the nuclear and cytosolic fractions of SFN-treated hLECs for 24 h using immunoblotting (Figure 2B). Immunoblot results using Nrf2 or Klf9 antibodies revealed that Nrf2 or Klf9 migrated at approximately 110 kDa or 32 kDa on SDS-PAGE(sodium dodecyl sulphate-polyacrylamide gel electrophoresis). Enrichment of both molecules in nuclear extracts of hLECs was increased with increases of SFN dose, and maximum accumulation was observed at 24 μM of SFN (Figure 2B, I and II). This increased nuclear accumulation of Nrf2/Klf9 was directly related to reduction in the cellular abundance of Prdx6 protein, indicating that Klf9 may be involved in suppression of Prdx6 in hLECs treated with higher doses of SFN, as reported previously in another model system.

We found a dramatic inverse correlation between expression of *Nrf2* and its target gene *Prdx6* transcripts in cells treated with higher doses of SFN (Figure 2A), which was unexpected, as Nrf2 is a transactivator of Prdx6. We postulated that the inverse correlation may be related to dysregulation of DNA binding efficiency/activity in Nrf2, or other adverse signaling may be involved. To investigate that possibility, we examined Nrf2 transactivation capacity. Nuclear fraction isolated from hLECs treated with increasing doses of SFN having equal amounts of protein was employed to quantify the activity of Nrf2 by TransAM *Nrf2* transcription factor assay (Active Motif) as shown in Figure 2C. Data revealed that Nrf2-DNA binding activity progressively increased with higher doses of SFN, indicating that DNA binding activity of Nrf2 was not attenuated by higher doses of SFN or SFN-induced increased ROS production. Collectively, these experiments indicate that some other factor may be involved in the repression of Prdx6 transcription. Since Prdx6 was suppressed at mRNA levels, we focused our further experimentation on transcriptional mechanisms by conducting in silico analysis of *Klf9* and *Prdx6* promoters for putative binding sites of Nrf2 and Klf9, respectively.

### 3.3. Bioinformatics Analyses Revealed Putative Nrf2 Activating and Klf9 Repressive Binding Sites in Klf9 (A) and Prdx6 (B) Promoters, Respectively

**Figure 3:**
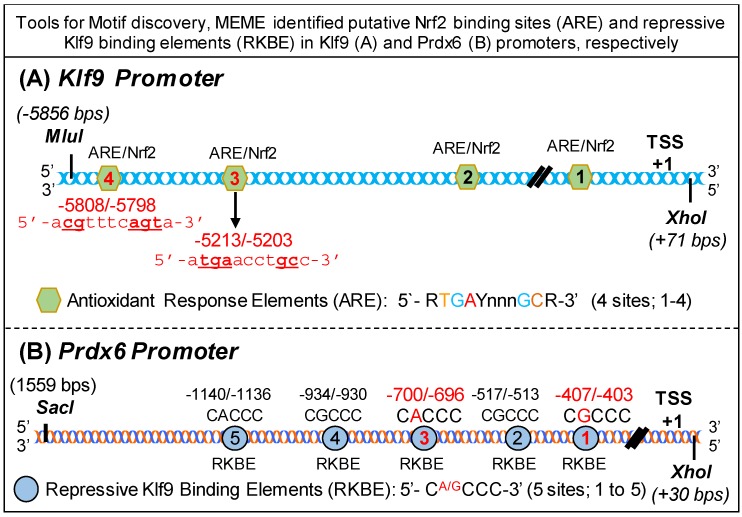
Diagrammatic sketch representing in silico analysis of putative Nrf2 and Klf9 binding sites in *Klf9* and *Prdx6* promoters, respectively. A tool for Motif discovery, MEME (Multiple Expectation maximization for Motif Elicitation), was used to spot out putative Nrf2 binding sites (ARE) and repressive Klf9 binding elements (RKBE) in *Klf9* (**A**) and *Prdx6* (**B**) promoters, respectively. *Klf9* promoter bears four Nrf2/ARE (5′-RTGAYnnnGC-3′) sites, as indicated in Figure 3A. *Prdx*6 promoter bears five Klf9 repressive binding sites (5′-C^A/G^CCC-3′), as indicated in Figure 3B.

To study the molecular mechanism underlying suppression of Nrf2/ARE-mediated antioxidant protective pathway in hLECs treated with higher doses of SFN, we utilized tools for Motif discovery, MEME. This in silico analysis identified putative Nrf2 binding sites and repressive Klf9 binding sites in Klf9 (Figure 3A) and hPrdx6 (Figure 3B) promoters, respectively. We found that *Klf9* promoter, spanning from 5′ −5856 to +1 bp (TSS), contained four well-conserved antioxidant response elements (ARE, 5′-R*G*GAYnnnGC-3′) [13,26], a known binding site for transcription factor, Nrf2 (Figure 3A). Intriguingly, analysis of *Prdx6* promoter, ranging from 5′-1559 to transcription start site (+1) bp, showed that it had five conserved Repressive Klf9 Binding Elements (RKBE; 5′-C^A/G^CCC-3′) (Figure 3B) in the regulatory region of *Prdx6* promoter. Moreover, the presence of a functional Nrf2/ARE binding site in *Prdx6* promoter has been reported by our group and others [1,82].

### 3.4. In Vivo DNA and Protein Interaction Disclosed that Klf9 Was Transcriptional Target for Nrf2, and SFN Treatment Enhanced Nrf2/ARE Binding in Dose-Dependent Fashion

**Figure 4:**
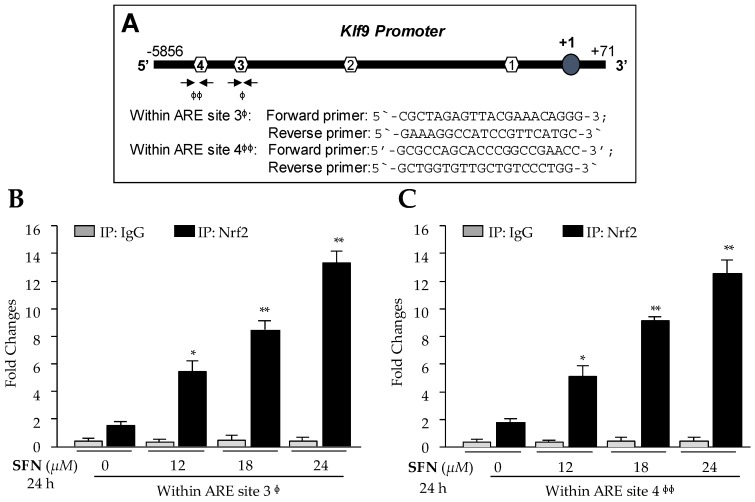
SFN induced increased Nrf2 binding to ARE sites in *Klf9* promoter in dose dependent fashion. (**A**) Top panel explaining the presence of four ARE sites in *Klf9* promoter and ChIP-qPCR primers containing ARE3 and ARE4. (**B** and **C**) An in vivo DNA binding assay revealed Nrf2/ARE interaction. hLECs treated with DMSO control or different concentration of SFN (0, 12, 18 and 24 µM) for 24 h, as indicated. ChIP assay was carried out with Nrf2 and control IgG antibodies. Pulled DNA fragments were subjected to qPCR analysis. DNA fragments present in the immunoprecipitation were amplified with primers that specifically recognize a fragment of *Klf9* promoter containing ARE sites as indicated. As a negative control, ChIP with control IgG was used. * *p* < 0.05, ** *p* < 0.001; SFN treated versus DMSO control. Φ and ΦΦ denotes primer pairs used for ChIP-qPCR. Note: Predicted sites ARE1 and ARE2 by in silico analysis are false positive sites (current study and [26]).

*Nrf2* is not a bona fide regulator of Klf9, as are antioxidant genes. However, it has been shown that *Klf9* gene is transcriptionally regulated by Nrf2 during higher levels of oxidative stress [26]. Given the significant induction of Klf9 mRNA and increased nuclear localization of Nrf2 with higher doses of SFN in hLECs, we sought to validate the previous findings [26] and determine whether Nrf2 directly bound to ARE sites present in regulatory region of *Klf9* promoter as predicted (Figure 3A) in hLECs treated with cytotoxic doses of SFN. We carried out ChIP assay to confirm the occupancy of Nrf2 on ARE of *Klf9* gene promoter as designated in Figure 4A. Human LECs treated with SFN (DMSO or 12–24 µM) for 24 h were processed for ChIP assay using antibody specific to Nrf2 or control IgG antibodies as described in Materials and Methods. The immunoprecipitated products were processed for RT-qPCR with primers flanking Nrf2 binding sites (Sites 3 and 4) in Klf9′s regulatory regions (Figure 4A). As shown in Figure 4B,C, we observed a progressive increase in enrichment of Nrf2 on both sites, ARE 3 and ARE 4 sequences, and found that enrichment was SFN dose-dependent, suggesting that higher doses of SFN (beyond survival doses) resulted in the aberrant and unphysiological accumulation of Nrf2 in nucleus, leading to Nrf2 binding to ARE of *Klf9* promoter as evidenced by ChIP-qPCR (Figure 4B,C, Black bars). Unexpectedly, Nrf2 failed to bind to predicted sites 1 and 2 (Figure 4A, data not shown), indicating that these sequences may be not be active sites. No binding was detected with control IgG (Figure 4B,C, gray bars), validating the specificity of Nrf2 antibody used in the experiments. These data indicated that higher doses of SFN augmented enrichment of Nrf2 at ARE sequences, and may be the molecular mechanism underlying upregulation of Klf9 in hLECs treated with toxic doses of SFN, if Nrf2/ARE binding was functional.

### 3.5. Higher Doses of SFN Activated Klf9 Transcriptional Activity through Nrf2/ARE Interaction 

**Figure 5:**
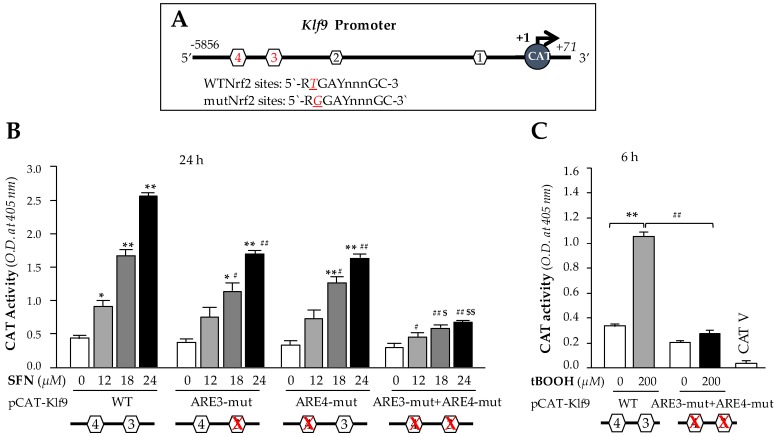
SFN activated *Klf9* transcriptional activity through Nrf2 in dose dependent manner. (**A**) Top panel; diagrammatic illustration of *Klf9* promoter containing predicted ARE sites with wild type (WT) ARE and mutant sequence (mutated using SDM as described in Materials and Methods). (**B** and **C**) hLECs were transfected with wild-type *Klf9* promoter (−5856/+71) or its mutant at ARE3 (ARE3-mut) or at ARE4 (ARE4-mut) and at both ARE3 plus ARE4 (ARE3-mut+ARE4-mut) sites. After 48 h, transfectants were treated with DMSO control or different concentration of SFN (12, 18 and 24 µM) for 24 h (**B**) and tBOOH (200 µM) for 6 h (**C**) as shown, and *Prdx6* Promoter activity was measured. * *p* < 0.05, ** *p* < 0.001; SFN treated versus untreated control, ^#^ *p* < 0.05, ^##^ *p* < 0.001; ARE3-mut or ARE4-mut or ARE3-mut+ARE4-mut versus WT, ^$^ *p* < 0.05, ^$$^ *p* < 0.001; ARE3-mut+ARE4-mut versus ARE3-mut or ARE4-mut.

We next sought to determine whether the observed Nrf2 binding to ARE present in the regulatory region that occurred following excessive doses of SFN in hLECs was functional and could activate Klf9 transcription. The results from in vivo DNA binding (Figure 4) led us to engineer four CAT reporter plasmids of Klf9 promoter: (i) pCAT-Klf9 wild-type (WT); (ii) pCAT-Klf9 mutant (mut) at site 3 (ARE3-mut, *T*GAYnnnGC; *T* to G); (iii) pCAT-Klf9 mutant at site 4 (ARE4-mut; *T* to G) (iv) pCAT-Klf9 mutant at both sites (ARE3-mut + ARE4-mut). To examine the functional consequences of SFN-induced changes on *Klf9* transcription, we transfected hLECs with a pCAT-Klf9 WT reporter plasmid (ARE; RTGAYnnnGC) or its mutant plasmids (RGGAYnnnGC) as noted above, along with a GFP plasmid as indicated in Figure 5. These transfectants were treated with SFN (DMSO or 12–24 µM) for 24 h. We found that the transactional activity of mutant plasmids mutated at ARE was significantly reduced compared to wild-type plasmids in response to increasing amount of SFN (Figure 5B); however, plasmids having mutation at both ARE sites of *Klf9* promoter showed robust loss in transcription activity compared to wild-type promoter′s transcriptional activity in response to higher doses of SFN (Figure 5B). Together, data showed that both ARE sites of *Klf9* promoter played a part in activation of *Klf9* transcription in response to higher doses of SFN. It was interesting to observe that SFN failed to activate *Klf9* transcription at lower concentrations (data not shown) at which SFN activates Nrf2-mediated antioxidant pathway [1]. We surmised that ARE3 and ARE4 of Klf9 have lower affinity than ARE of *Prdx6* promoter; however, exploring this phenomenon would require a separate line of investigation. Furthermore, to determine whether upregulation of Klf9 is activated only by SFN, or whether other Nrf2 activators at high doses may have the same effect, we treated transfectants containing wild-type pCAT-Klf9 and its ARE mutant at both sites for 6 h with a high dose of tBOOH (200 μM), a well-known activator of Nrf2 [83]. Similar to SFN, treatment with the high dose of tBOOH resulted in a robust increase in *Klf9* transcriptional activity, in contrast to mutant plasmid (Figure 5C), suggesting that the upregulation of Klf9 is not specific to SFN, but can occur with other Nrf2 activators applied at higher or cytotoxic doses.

### 3.6. Treatment of hLECs with Increased Amounts of SFN Mobilized the Klf9 Enrichment on Its Binding Sites Present in Prdx6 Promoter

**Figure 6:**
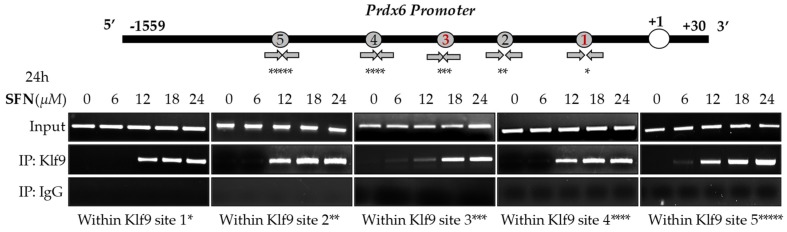
Higher doses of SFN enforced Klf9 binding to the regulatory region of its new target gene, *Prdx6*. Top panel; diagrammatic illustration of in silico analysis of *Prdx6* gene promoter showing five putative repressive Klf9 binding elements (RKBE; nC^A/G^CCCn). hLECs treated with DMSO or different concentration of SFN for 24 h, as indicated. Pulled DNA fragments with antibodies specific to Klf9 were subjected to PCR analysis for repressive Klf9 binding elements (RKBE) as described in Materials and Methods. DNA fragments present in the immunoprecipitates were amplified with primers that recognized a fragment of *Prdx6* promoter containing RKBE as indicated. Control IgG served as negative control. Primers used for amplification of specific region containing Klf9 sites (* Klf9 site 1, ** Klf9 site 2, *** Klf9 site 3, **** Klf9 site 4, ***** Klf9 site 5). Primer sequences were mentioned in the Materials and Methods section.

In silico analyses spotted out the presence of five RKBEs (a single GC boxes) [26,33] in *Prdx6* promoter. Recent report has shown that aberrant expression of *Klf9* and nuclear accumulation leads to repression of antioxidant genes [26]. Based on this information, we posit if the observed inverse correlation between Prdx6 and aberrant expression and increased accumulation of *Klf9* (Figure 2) in response to lethal doses of SFN can be connected to repression of Prdx6 transcription in hLECs; thus, we carried out an in vivo DNA binding experiment. We treated hLECs with increasing amounts of SFN (6 to 24 μM) and performed ChIP assay to measure the occupancy of *Klf9* at *Prdx6* gene promoter (1559 bp upstream of it transcription start site). As shown in Figure 6, Klf9 occupied the *Prdx6* promoter at RKBEs (5′-C^A/G^CCC-3′), and the increased enrichment of Klf9 to the sequences was SFN concentration-dependent, while no enrichment of Klf9 occurred at 6 µM of SFN. Moreover, no amplicon was detected with control IgG, validating the specificity of Klf9 antibody used in the experiment. Thus, the experiment revealed that Klf9 interacted with all RKBEs of *Prdx6* promoter in vivo as predicted through bioinformatics tool, and the progressive increase in the binding of Klf9/RKBE was SFN dose-dependent, as indicated in Figure 6.

### 3.7. Transcriptional Assay Disclosed that Klf9/RKBE Interaction Transcriptionally Repressed Its New Target Gene, Prdx6 during High Doses of SFN

**Figure 7:**
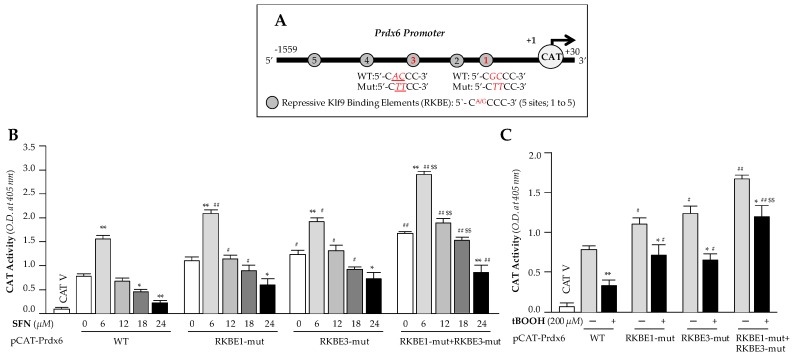
Transcription assay disclosed that Klf9 repressed *Prdx6* expression during high dose of SFN. (**A**) Diagrammatic illustration of *Prdx6* promoter containing RKBE sites predicted by in silico analysis and showing wild type (WT) and its mutant sequences used for promoter assay. (**B** and **C**) hLECs were transfected with Prdx6 (−1559 / + 30) wild type (WT) plasmid construct or its mutants at RKBE1 (RKBE1-mut) or at RKBE3 (RKBE3-mut) or RKBE1 plus RKBE3 (RKBE1-mut+ RKBE3-mut). After 48 h, transfectants were treated with different concentration of SFN for 24 h (**B**) and tBOOH (**C**) for 6 h, as indicated. Promoter activity is measured and data represent mean ± S.D. values of three independent experiments. * *p* < 0.05, ** *p* < 0.001; SFN treated versus untreated control, ^#^ *p* < 0.05, ^##^ *p* < 0.001; RKBE1-mut or RKBE3-mut or RKBE1-mut+ RKBE3-mut versus WT, ^$$^ *p* < 0.001; RKBE1-mut+ RKBE3-mut versus RKBE1-mut or RKBE3-mut.

To examine the functionality of the dose-dependent SFN-induced increased Klf9 binding to RKBE on Prdx6 transcription, we performed transactivation assay in hLECs containing *Prdx6* promoter fused to reporter gene CAT, and treated cells with the same increasing doses of SFN as shown in Figure 6. We constructed *Prdx6* promoter linked to CAT reporter gene plasmid having Klf9 sites (Figure 7A), and mutated sites, RKBE1, RKBE3 and RKBE1 + RKBE3 and mutated them using site directed mutagenesis (SDM) to test the functionality of binding shown in Figure 6. pCAT-Prdx6 Wild type (WT) or its mutants at RKBE1 (RKBE1-mut) or RKBE3 (RKBE3-mut) or at both sites (RKBE1-mut+ RKBE3-mut) of *Prdx6* promoter plasmids were transfected into hLECs. 48h later transfectants were treated with different concentrations of SFN (for 24h) or tBOOH (6h) as shown in Figure 7. Transcriptional activity of pCAT-Prdx6 WT was dramatically reduced with increasing doses of SFN treatment, but was significantly restored in mutant plasmids, and pCAT-Prdx6-RKBE1-mut plus RKBE3-mut plasmid showed a further increase in transcriptional activity than pCAT-Prdx6- RKBE1-mut or RKBE3-mut as indicated in Figure 7. Those observations suggest that observed Klf9/RKBE binding in *Prdx6* promoter (Figure 6) were functional, and loss of *Prdx6* expression in hLECs treated with toxic doses of SFN was caused by Klf9/RKBE present in regulatory region of *Prdx6* promoter. Next, we tested the effect of another known Nrf2 activator, where tBOOH acts through ARE, on the transcriptional activity of Prdx6. Similar to the SFN experiments noted above, transfectants containing mutant reporter plasmids mutated either at one RKBE site (RKBE1-mut or RKBE3-mut) or both sites (RKBE1-mut plus RKBE3-mut) and provided a similar pattern of Prdx6 transcription as observed in SFN experiments in cells treated with toxic dose of tBOOH (200μM for 6h) (Figure 7C, gray bars versus black bars), showing a significant revival of transcriptional activity in mutant *Prdx6* promoters mutated at RKBE as seen in Figure 7C. Nevertheless, data demonstrated that mutation at the above-noted two sites did not completely eliminate SFN-driven Klf9-mediated suppression of *Prdx6* promoter activity, pointing to the contribution of three other sites (RKBE2, RKBE4 and RKBE5) in Prdx6 repression. Collectively, data revealed that RKBE negatively regulated *Prdx6* expression through repressing its transcription in cells facing SFN-induced toxicity. This also suggests that the advent of Klf9-mediated dominant repressive signaling in hLECs in response to high doses of SFN that surpasses Nrf2-mediated activation of Prdx6. Results unveiled the molecular mechanism involved in SFN-mediated cytotoxicity, which is linked to attenuation of Prdx6 by Nrf2/Klf9 axis as shown in Figure 1 and Figure 2.

### 3.8. Klf9-Deficient hLECs Showed Resistance against Treatment with Toxic Doses of SFN

**Figure 8:**
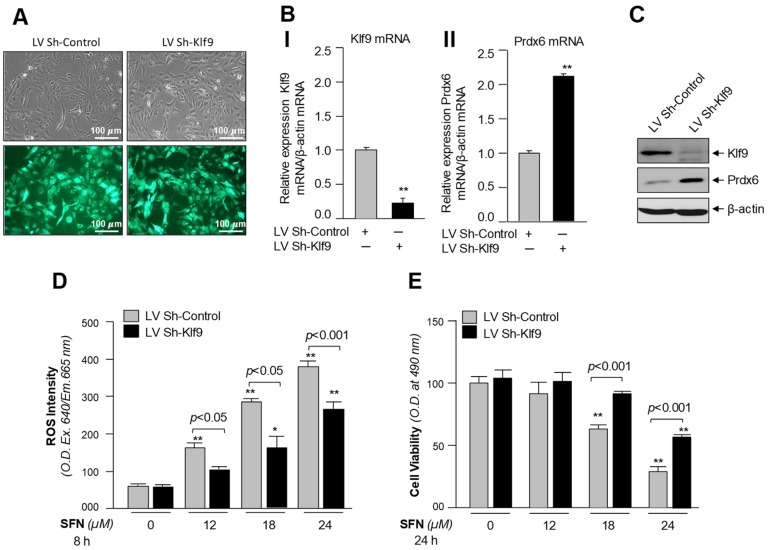
Klf9-deficient hLECs displayed increased *Prdx6* expression with reduced ROS level and conferred resistance against toxic doses of SFN. (**A**–**C**) Klf9- knockdown with its ShRNA showed enhanced *Prdx6* expression. hLECs were stably infected either with GFP (green fluorescent protein) linked lentiviral (LV) Sh-Control or GFP linked LV Sh-Klf9 linked as described in Materials and Methods. (**A**) Photomicrograph representing stably infected hLECs, left panel: LV Sh-Control; right Panel: LV Sh-Klf9. (**B**) Total RNA was isolated from infected hLECs and RT-qPCR analysis was conducted with primers specific to Klf9 and Prdx6. Data represent the mean ± S.D. of three independent experiments. ** *p* < 0.001; LV Sh-Klf9 versus LV Sh-Control. (**C**) Cellular extract prepared from infected hLECs having equal amounts of protein was immunoblotted with antibody specific to Klf9 or Prdx6 or β-actin. (**D** and **E**) Depletion of Klf9 suppressed ROS levels and increased the cell viability. hLECs were stably infected with LV Sh-Control or LV Sh-Klf9 lentiviral particle following section pressure as described in Materials and Methods. Cells were harvested in 96 well plate. After24 h, those cells were exposed to DMSO (0), 12, 18 and 24 μM of SFN. 8 h of SFN exposure ROS intensity (**D**) and at 24 h cell viability (**E**) were examined. * *p* < 0.05, ** *p* < 0.001; SFN treated versus control, *p* < 0.05, *p* < 0.001; LV Sh-Klf9 versus LV Sh-Control.

SFN is a major activator with diversified functions, including a cytoprotective effect. Its protective activity depends on the amount of SFN applied to cells and on cell types [1,63,67]. Results from the current study revealed that the Nrf2/Klf9 axis played an adverse role by repressing the protective antioxidant gene *Prdx6* in response to high doses of SFN. Recently, several reports have noted that SFN application can halt or abate several diseases and their complications [1,84,85,86,87,88,89,90,91,92,93], indicating that a dose of SFN is indispensable to Nrf2/ARE-driven protective pathway. Because of its effectiveness in cytoprotection and the cytotoxicity of its higher doses via aberrant expression of *Klf9*, we used a Klf9 knockdown strategy to examine if Klf9-depleted cells can resist increasing doses of SFN. Our goal was to provide a proof of concept for a potential basis for combination therapy (SFN plus Klf9-ShRNA). As described in Materials and Methods, we stably infected hLECs using lentiviral (LV) Sh-Klf9 or LV Sh-control (Figure 8A), and examined the effect of Klf9 depletion on SFN-mediated cytotoxicity. As shown in Figure 8B(I,II),C, RT-qPCR and immunoblot analyses revealed that LV Sh-Klf9 significantly knocked down the *Klf9* expression in hLECs infected with LV Sh-Klf9. It was intriguing to observe that deficiency of Klf9 was directly linked to upregulation of Prdx6 mRNA and protein, corroborating that Klf9 has played a part in repression of *Prdx6* expression. Next, we treated these tranfectants with increasing toxic doses of SFN for 24 h (Figure 8D,E) and assessed the levels of ROS and cell survival. ROS assay and viability assay revealed that toxic doses of SFN were significantly less effective at inducing ROS-evoked toxicity in Klf9-deficient cells, and survival of these cells was increased (Figure 8E, black bars versus gray bars) with significant reduction in ROS levels, demonstrating that aberrant expression of *Klf9* was a major cause of SFN-mediated cytotoxicity. Thus, we propose that SFN application along with Klf9, ShRNA should be considered as a novel strategy to treat Nrf2/ARE dysregulation-mediated diseases during aging or oxidative stress.

## 4. Discussion

The redox stress-sensitive, Nrf2-antioxidant response element (ARE)-mediated antioxidant pathway is the major cellular defense against several internal and external environmental stresses. Oxidative stress and many compounds with electrophilic properties, such as heavy metals, tBOOH and naturally occurring phytochemicals such as SFN, have been reported to yield therapeutic benefits by modulating Nrf2-ARE pathway [1,27,67,94,95,96,97,98]. At physiological conditions, Nrf2 is controlled via Keap1, an adopter protein for CULLI3-based ubiquitin E3 ligase that ubiquitinates Nrf2 for proteasomal degradation. During exposure to electrophiles (such as SFN) Keap1 is inactivated due to binding of SFN and that subsequently leads to dislodging of Nrf2 from Keap1, and allows Nrf2 to escape from degradation and translocalizes into the nucleus, where it activates antioxidant genes [5,99]. In recent years, several studies have shown that SFN has a cytoprotective function with diversified activities [26,63]. Nevertheless, studies have shown that SFN does not act as direct antioxidant, but acts via upregulating the Nrf2/ARE pathway [27,100]. SFN may also induce the phosphorylation of Nrf2 by activating various kinases, where it can alter nuclear and cytoplasmic trafficking as well as Nrf2 integrity [101]. In maintaining redox-homeostasis in the cellular microenvironment, *Nrf2* expression and activity are tightly regulated [21]. However, aberrant expression and activation of *Nrf2*, in response to increased doses of electrophiles or oxidative load, are known to induce adverse signaling, failure of redox homeostasis and cell death [1,26,63,102,103,104]. This suggests that the diversified activity of Nrf2/ARE-mediated response is connected to its regulation in the cellular microenvironment and doses of Nrf2 activators; at low doses, most effectors of Nrf2/ARE pathway provide cytoprotection and higher doses are cytotoxic under the conditions of cell background and treatment regimens. However, studies of the molecular mechanisms involved in the dose-dependent switch from cell survival to cell death have been limited, and the mechanisms have not been investigated in lens cells. In recent work, we observed that increase amounts of SFN induced cytotoxicity, while nontoxic doses enhanced cell survival by activating Nrf2/ARE/Prdx6 pathway (current study and [1]). However, higher doses of SFN are known to cause cell death in various cell types and to inhibit tumor cell growth [98,105,106,107,108]. Using hLECs and Nrf2/Prdx6 axis as a model system, in the present study, we found that toxic doses of SFN dramatically enhanced *Nrf2* expression and its nuclear accumulation, which was correlated with increased expression and nuclear accumulation of *Klf9* and reduced expression of *Prdx6* transcripts and protein (Figure 1 and Figure 2). We thought this might be associated with reduced DNA binding efficiency of Nrf2 to *Prdx6* gene promoter [1,9,109], but experimentation ruled out that possibility. Conversely, data showed that Nrf2′s DNA binding efficacy increased with increased doses of SFN (Figure 2C). Zucker et al. [26] demonstrated that over-accumulation of Nrf2 in nucleus targets Klf9 and significantly upregulates its transcription by binding to ARE present in *Klf9* promoter, which in turn binds to GC box sequences present in some of antioxidant genes and represses their transcription. Intriguingly, in silico analysis of *Prdx6* gene promoter (Figure 3B) coupled with DNA binding and transactivation assays disclosed that repression of Prdx6 is linked to Klf9 in cells treated with increased amounts SFN (Figure 4, Figure 5, Figure 6, Figure 7 and Figure 8), emphasizing Klf9-mediated repressive signaling is dominant over Nrf2-mediated activation of Prdx6 in response to toxic doses of SFN. Klf9 is a ubiquitously expressed protein, with functions, which have been little explored in lens cells. Our data indicated that Klf9 could cause transcription repression of *Prdx6* gene in hLECs exposed to toxic amount of SFN. Prdx6 is a powerful antioxidant protective protein, which plays a critical role in the antioxidant defense system [1,7,43,44,52,57,58,72,73,76,77,78,79]. Prdx6 protects cells by regulating ROS levels and thereby maintains cellular homeostasis [1,52]. Prdx6 is abundantly expressed in eye, lungs, brain and other organs [1,6,7,56,57,58,79]. The protein maintains cell integrity by reducing peroxidized phospholipids in cell membrane, protecting DNA damage and controlling survival signaling during aging and oxidative stress [110]. Prdx6 localizes in almost all ROS producing organelles, such as mitochondria, endoplasmic, lysosome and plasma membrane, suggesting its importance in the cellular antioxidant defense system. 

In a previous publication, we reported that SFN, a known classic activator of Nrf2, enhances antioxidant proteins such as Prdx6, catalase and GST*π* in human, mouse and rat LECs in a concentration-dependent manner [1]. Other studies found that pretreatment of mouse fibroblasts or LECs with nontoxic concentrations of SFN (3–6 µM) increased their resistance against oxidative stress caused by paraquat, hydrogen peroxide or UVB (ultra violet- B) in an Nrf2-dependent manner [1,81]. As shown in Figure 1B, a nontoxic (6 µM) concentration of SFN enhanced hLECs viability, while toxic/lethal concentrations (12–24 µM) of SFN caused cell death that was directly associated with increased ROS generation (Figure 1A, black bars). Doses above 10 µM of SFN concentration has been reported to reduce cell viability and cause cell death in FHL124 cells [111]. Up to 10 µM of SFN enhances the viability of N9 cells, while 20 µM and 50 µM concentrations increase cell death [112]. Moreover, toxic doses of SFN have been shown to increase Nrf2 activity [112] leading to Klf9 upregulation. Our current data is consistent with a previous report [26] revealing that increased nuclear abundance of Nrf2 is connected to increased Klf9 levels and reduced expression of *Prdx6* mRNA and protein in hLECs in response to high doses of SFN (Figure 2A,B). SFN has been found to enhance Nrf2/ARE-mediated antioxidant genes mRNA and protein expression in cells from C57BL/6J mice and humans, and in mouse, rat and human LECs [1,113]. SFN at high concentrations is known to cause cell death due to oxidative stress [26,98]. In this work, we sought to determine whether similar Nrf2/Klf9-mediated injurious signaling occurs in lens cells in treated with increased doses of SFN. Published reports and the current study have shown that low doses of SFN increase the levels of antioxidant molecules without inducing Klf9 [81]. A Klf9-dependent feed-forward regulatory loop has been shown to trigger ROS-evoked cell death [114,115]. Collectively, our data revealed a model of Klf9-dependent feed-forward regulation of ROS that occurred through Nrf2 upregulation of Klf9 and Klf9-mediated repression of Prdx6 in LECs treated with increased amount of SFN. At low concentrations of SFN, Nrf2-dependent antioxidant is activated, but at higher concentrations, Nrf2 continues to accumulate in the nucleus, leading to Nrf2 binding to the *Klf9* promoter, increased *Klf9* transcription, and further increases in ROS levels (Figure 1 and Figure 8). Previously, we have reported that Prdx6 depletion causes increased vulnerability to oxidative stressors-induced cell death. In addition, we observed that other cellular antioxidants were ineffective at protecting LECs [73,76,116], demonstrating the importance of Prdx6 for cellular survival and cytoprotection. Nrf2 activation of Prdx6 provides cytoprotection by limiting ROS. Intracellular ROS are generated in cells by nonenzymatic and enzymatic process such as superoxide-dismutase (SOD)-catalyzed disproportionation of the superoxide radicals to H_2_O_2_ and by redox cycling. We believe that exposure of a toxic dose of SFN or oxidants can contribute substantially to the cellular steady levels of H_2_O_2_ and the super oxide radicals due to reduced availability of Prdx6 caused by Nrf2/Klf9 axis-mediated Prdx6 suppression. Recently, it has been reported that Nrf2 can modulate ROS generation [117]. However, how redox balance is maintained following electrophiles exposure is cumbersome to explain at this time point, and thus, further work is warranted. Nonetheless, constitutive production of H_2_O_2_ is derived from mitochondria dependent on nicotinamide adenine dinucleotide (NADH) [118] and activity of nicotinamide adenine dinucleotide phosphate (NADPH) oxidase (NOX) enzymes dependent upon NADPH [119,120]. Aberrant activity of these enzymes has been suggested to contribute to the onset of many diseases linked to oxidative stress. Prdx6 is localized in ROS-producing organelles, such as mitochondria, endoplasmic reticulum and peroxisomes, indicating that Prdx6 deficiency in these organelles can disorient ROS-mediated survival signaling leading to oxidative stress-induced cellular damage or cell death. Furthermore, recently, *Klf9* has been identified as a new target gene for Nrf2 [26,121,122]. The *Klf9* regulatory region bearing active ARE3 and ARE4 (Figure 3A, Figure 4 and Figure 5) has lesser affinity for *Nrf2* than the ARE present in antioxidant genes [26]. We observed similar phenomena, as Figure 4 and Figure 5 show increased functional binding of Nrf2 to ARE of Klf9 in response to lethal doses of SFN.

**Figure 9:**
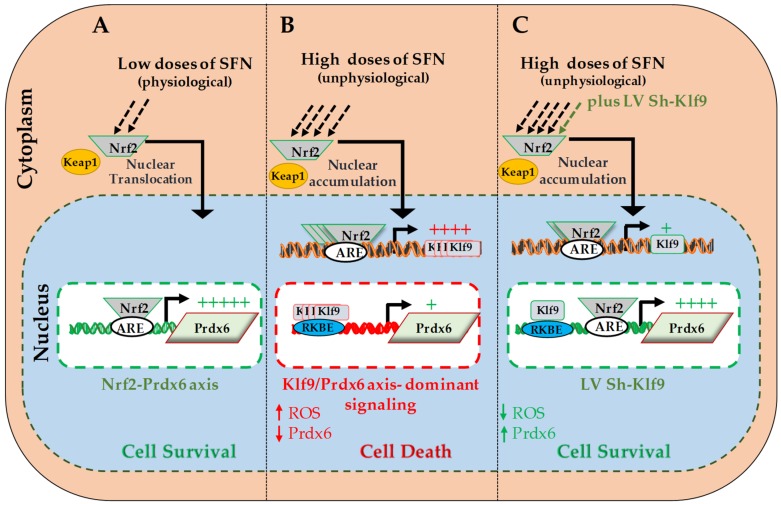
Diagram illustrating a plausible molecular mechanism underlying the SFN evoked Nrf2/Klf9/Prdx6 axis that determines cell fate. Role of Nrf2/Klf9/Prdx6 axis in response to SFN. (**A**) At physiological level Nrf2-Keap1 complex resides in cytoplasm. With nonlethal doses of SFN (electrophilic stress), Nrf2 translocalizes into nucleus, binds to ARE in regulatory region of antioxidant genes like *Prdx6* gene promoter, and upregulates their transcription to maintain redox homeostasis and defend cells from stressors. (**B**) At high (lethal) doses of SFN, excess Nrf2 accumulates in the nucleus and induces expression of transcription factor Klf9 via ARE site(s) present in its promoter. *Klf9* represses *Prdx6* expression by binding to repressive binding element(s) (RKBE) in the *Prdx6* gene promoter, resulting in increased ROS-mediated cell death. (**C**) Klf9 can be depleted by use of its Sh-Klf9, eliminating the adverse signaling caused by lethal doses of SFN. This suggests that Klf9 upregulation is an important molecular mechanism underlying SFN-induced ROS-mediated cell injury.

Klf9 belongs to the KLF transcription factor family and has diversified roles in oncogenesis, cell differentiation and development and cell death [26,38]. Klf9 acts as both a transcription activator and repressor, and in doing so, controls gene expression. Klf9 activates promoters having repeated GC boxes like Sp1, but represses transcription of genes containing a single GC box sequence [33]. Our in vivo DNA-binding and transactivation assays revealed that, indeed, *Klf9* acted as repressor of *Prdx6* expression. We found that *Prdx6* genes bear five *Klf9* repressive binding sites (RKBE; C^A/G^CCC), and these sites were not repeated GC boxes, but were single GC box sequences, demonstrating that Klf9 acted as repressor for Prdx6 by binding to RKBE 1, 2, 3, 4 and 5 present in its gene promoter during toxic doses of SFN (Figure 6 and Figure 7). Interestingly, using knockdown assay, we found that Klf9 deficiency in hLECs enhanced *Prdx6* expression and reduced levels of ROS, and these cells survived well in response to increased doses of SFN (Figure 8). These data reveal the molecular mechanism of SFN-mediated diversified function, and demonstrate that Klf9′s cellular level is the major factor determining dose-dependent SFN-mediated activity. Furthermore, SFN has been shown to influence the activity and expression of various transcription factors such as NF-кB [123], Ap1 [124], Sp1 [125,126] and Nrf2 [127] and so forth, by modulating cellular signaling, including antioxidant expression. However, our study found that increased ROS-induced toxic doses of SFN play a part in repression of Prdx6 via the Nrf2/Klf9 axis as shown in Figure 9.

## 5. Conclusions

In conclusion, our work revealed, for the first time, the novel molecular mechanism underlying how SFN-induced Nrf2-mediated protective signaling goes awry in hLECs in response to increased doses of Nrf2 activator, SFN. We showed that during increased doses of SFN, Klf9-mediated repression of *Prdx6* expression is a major event, resulting in acceleration of ROS accumulation and cell death. Thus, dose levels of SFN determine whether it has beneficial or harmful effects, with Nrf2/Klf9/Prdx6 axis being pivotal for SFN activities. Based upon our data, it should be of prime importance to evaluate doses of SFN or other Nrf2 activators, which do not evoke Klf9/Prdx6 axis (-mediated adverse signaling). We propose that a therapy combining SFN with a Klf9 ShRNA would be an ideal approach to abating or treating disorders linked to aging and oxidative stressors.

## References

[1] Kubo E., Chhunchha B., Singh P., Sasaki H., Singh D.P. (2017). Sulforaphane reactivates cellular antioxidant defense by inducing Nrf2/ARE/Prdx6 activity during aging and oxidative stress. Sci. Rep..

[2] Tonelli C., Chio I.I., Tuveson D.A. (2018). Transcriptional Regulation by Nrf2. Antioxid. Redox Signal..

[3] Halliwell B. (2007). Biochemistry of oxidative stress. Biochem. Soc. Trans..

[4] Schieber M., Chandel N.S. (2014). ROS function in redox signaling and oxidative stress. Curr. Boil..

[5] Itoh K., Wakabayashi N., Katoh Y., Ishii T., Igarashi K., Engel J.D., Yamamoto M. (1999). Keap1 represses nuclear activation of antioxidant responsive elements by Nrf2 through binding to the amino-terminal Neh2 domain. Genes Dev..

[6] Chhunchha B., Fatma N., Kubo E., Rai P., Singh S.P., Singh D.P. (2013). Curcumin abates hypoxia-induced oxidative stress based-ER stress-mediated cell death in mouse hippocampal cells (HT22) by controlling Prdx6 and NF-kappaB regulation. Am. J. Physiol. Cell. Physiol..

[7] Chhunchha B., Fatma N., Bhargavan B., Kubo E., Kumar A., Singh D.P. (2011). Specificity protein, Sp1-mediated increased expression of Prdx6 as a curcumin-induced antioxidant defense in lens epithelial cells against oxidative stress. Cell Death Dis..

[8] Thimmulappa R.K., Mai K.H., Srisuma S., Kensler T.W., Yamamoto M., Biswal S. (2002). Identification of Nrf2-regulated genes induced by the chemopreventive agent sulforaphane by oligonucleotide microarray. Cancer Res..

[9] Suh J.H., shenvi S.V., Dixon B.M., Liu H., Jaiswal A.K., Liu R.-M., Hagen T.M. (2004). Decline in transcriptional activity of Nrf2 causes age-related loss of glutathione synthesis, which is reversible with lipoic acid. Proc. Natl. Acad. Sci. USA.

[10] Poprac P., Jomova K., Simunkova M., Kollar V., Rhodes C.J., Valko M. (2017). Targeting Free Radicals in Oxidative Stress-Related Human Diseases. Trends Pharmacol. Sci..

[11] Wasserman W.W., Sandelin A. (2004). Applied bioinformatics for the identification of regulatory elements. Nat. Rev. Genet..

[12] Wasserman W.W., Fahl W.E. (1997). Comprehensive Analysis of Proteins Which Interact with the Antioxidant Responsive Element: Correlation of ARE-BP-1 with the Chemoprotective Induction Response. Arch. Biochem. Biophys..

[13] Wasserman W.W., Fahl W.E. (1997). Functional antioxidant responsive elements. Proc. Natl. Acad. Sci. USA.

[14] Calabrese E.J., Baldwin L.A. (2001). Hormesis: U-shaped dose responses and their centrality in toxicology. Trends Pharmacol. Sci..

[15] Calabrese E.J., Baldwin L.A. (2003). Hormesis: The dose-response revolution. Annu. Rev. Pharmacol. Toxicol..

[16] Calabrese E.J. (2005). Cancer Biology and Hormesis: Human Tumor Cell Lines Commonly Display Hormetic (Biphasic) Dose Responses. Crit. Rev. Toxicol..

[17] Calabrese E.J. (2005). Historical blunders: How toxicology got the dose-response relationship half right. Cell. Mol. Boil..

[18] Calabrese E.J. (2005). Toxicological awakenings: The rebirth of hormesis as a central pillar of toxicology. Toxicol. Appl. Pharmacol..

[19] Calabrese E., Blain R. (2005). The occurrence of hormetic dose responses in the toxicological literature, the hormesis database: An overview. Toxicol. Appl. Pharmacol..

[20] Morroni F., Tarozzi A., Sita G., Bolondi C., Moraga J.M., Cantelli-Forti G., Hrelia P. (2013). Neuroprotective effect of sulforaphane in 6-hydroxydopamine-lesioned mouse model of Parkinson′s disease. Neurotoxicology.

[21] Tebay L.E., Robertson H., Durant S.T., Vitale S.R., Penning T.M., Dinkova-Kostova A.T., Hayes J.D. (2015). Mechanisms of activation of the transcription factor Nrf2 by redox stressors, nutrient cues, and energy status and the pathways through which it attenuates degenerative disease. Free. Radic. Boil. Med..

[22] Lee J.M., Li J., Johnson D.A., Stein T.D., Kraft A.D., Calkins M.J., Jakel R.J., Johnson J.A. (2005). Nrf2, a multi-organ protector?. FASEB J..

[23] Li J., Johnson D., Calkins M., Wright L., Svendsen C., Johnson J. (2005). Stabilization of Nrf2 by tBHQ confers protection against oxidative stress-induced cell death in human neural stem cells. Toxicol. Sci..

[24] Burton N.C., Kensler T.W., Guilarte T.R. (2006). In vivo modulation of the Parkinsonian phenotype by Nrf2. NeuroToxicology.

[25] Pajares M., Cuadrado A., Rojo A.I. (2017). Modulation of proteostasis by transcription factor NRF2 and impact in neurodegenerative diseases. Redox Boil..

[26] Zucker S.N., Fink E.E., Bagati A., Mannava S., Bianchi-Smiraglia A., Bogner P.N., Wawrzyniak J.A., Foley C., Leonova K.I., Grimm M.J. (2014). Nrf2 amplifies oxidative stress via induction of Klf9. Mol. Cell.

[27] Kim H.-J., Barajas B., Wang M., Nel A.E. (2008). Nrf2 activation by sulforaphane restores the age-related decrease of T(H)1 immunity: Role of dendritic cells. J. Allergy Clin. Immunol..

[28] Kobayashi M., Yamamoto M. (2005). Molecular Mechanisms Activating the Nrf2-Keap1 Pathway of Antioxidant Gene Regulation. Antioxidants Redox Signal..

[29] Kansanen E., Kuosmanen S.M., Leinonen H., Levonen A.-L. (2013). The Keap1-Nrf2 pathway: Mechanisms of activation and dysregulation in cancer. Redox Boil..

[30] Cox A.G., Gurusinghe S., Rahman R.A., Leaw B., Chan S.T., Mockler J.C., Murthi P., Marshall S.A., Lim R., Wallace E.M. (2019). Sulforaphane improves endothelial function and reduces placental oxidative stress in vitro. Pregnancy Hypertens..

[31] Parga J.A., Rodriguez-Perez A.I., Garcia-Garrote M., Rodriguez-Pallares J., Labandeira-Garcia J.L. (2018). Angiotensin II induces oxidative stress and upregulates neuroprotective signaling from the NRF2 and KLF9 pathway in dopaminergic cells. Free. Radic. Boil. Med..

[32] Mannava S., Zhuang D., Nair J.R., Bansal R., Wawrzyniak J.A., Zucker S.N., Fink E.E., Moparthy K.C., Hu Q., Liu S. (2012). KLF9 is a novel transcriptional regulator of bortezomib- and LBH589-induced apoptosis in multiple myeloma cells. Blood.

[33] Imataka H., Sogawa K., Yasumoto K., Kikuchi Y., Sasano K., Kobayashi A., Hayami M., Fujii-Kuriyama Y. (1992). Two regulatory proteins that bind to the basic transcription element (BTE), a GC box sequence in the promoter region of the rat P-4501A1 gene. EMBO J..

[34] Mitchell D.L., DiMario J.X. (2010). Bimodal, Reciprocal Regulation of Fibroblast Growth Factor Receptor 1 Promoter Activity by BTEB1/KLF9 during Myogenesis. Mol. Boil. Cell.

[35] Good K.L., Tangye S.G. (2007). Decreased expression of Krüppel-like factors in memory B cells induces the rapid response typical of secondary antibody responses. Proc. Natl. Acad. Sci. USA.

[36] Simmen R.C.M., Eason R.R., McQuown J.R., Linz A.L., Chatman L., Fujii-Kuriyama Y., Kang T.-J., Till S.R., Simmen F.A., Oh S.P. (2004). Subfertility, Uterine Hypoplasia, and Partial Progesterone Resistance in Mice Lacking the Krüppel-like Factor 9/Basic Transcription Element-binding Protein-1 (Bteb1) Gene. J. Boil. Chem..

[37] Cui A., Fan H., Zhang Y., Zhang Y., Niu D., Liu S., Liu Q., Ma W., Shen Z., Shen L. (2019). Dexamethasone-induced Krüppel-like factor 9 expression promotes hepatic gluconeogenesis and hyperglycemia. J. Clin. Investig..

[38] Ying M., Tilghman J., Wei Y., Guerrero-Cazares H., Quinones-Hinojosa A., Ji H., Laterra J. (2014). Kruppel-like factor-9 (KLF9) inhibits glioblastoma stemness through global transcription repression and integrin alpha6 inhibition. J. Biol. Chem..

[39] Bagati A., Moparthy S., Fink E.E., Bianchi-Smiraglia A., Yun D.H., Kolesnikova M., Udartseva O.O., Wolff D.W., Roll M.V., Lipchick B.C. (2019). KLF9-dependent ROS regulate melanoma progression in stage-specific manner. Oncogene.

[40] Arnér E.S. (2009). Focus on mammalian thioredoxin reductases—Important selenoproteins with versatile functions. Biochim. Biophys. Acta (BBA)-Gen. Subj..

[41] Itoh K., Chiba T., Takahashi S., Ishii T., Igarashi K., Katoh Y., Oyake T., Hayashi N., Satoh K., Hatayama I. (1997). An Nrf2/Small Maf Heterodimer Mediates the Induction of Phase II Detoxifying Enzyme Genes through Antioxidant Response Elements. Biochem. Biophys. Res. Commun..

[42] Gu Y., Wu Y.-B., Wang L.-H., Yin J.-N. (2015). Involvement of Kruppel-like factor 9 in bleomycin-induced pulmonary toxicity. Mol. Med. Rep..

[43] Kubo E., Miyazawa T., Fatma N., Akagi Y., Singh D.P. (2006). Development- and age-associated expression pattern of peroxiredoxin 6, and its regulation in murine ocular lens. Mech. Ageing Dev..

[44] Fatma N., Kubo E., Sen M., Agarwal N., Thoreson W.B., Camras C.B., Singh D.P. (2008). Peroxiredoxin 6 delivery attenuates TNF-alpha-and glutamate-induced retinal ganglion cell death by limiting ROS levels and maintaining Ca^2+^ homeostasis. Brain Res..

[45] Kümin A., Huber C., Rülicke T., Wolf E., Werner S. (2006). Peroxiredoxin 6 Is a Potent Cytoprotective Enzyme in the Epidermis. Am. J. Pathol..

[46] Kümin A., Schäfer M., Epp N., Bugnon P., Born-Berclaz C., Oxenius A., Klippel A., Bloch W., Werner S. (2007). Peroxiredoxin 6 is required for blood vessel integrity in wounded skin. J. Cell Biol..

[47] Manevich Y., Fisher A.B. (2005). Peroxiredoxin 6, a 1-Cys peroxiredoxin, functions in antioxidant defense and lung phospholipid metabolism. Free. Radic. Boil. Med..

[48] Manevich Y., Sweitzer T., Pak J.H., Feinstein S.I., Muzykantov V., Fisher A.B. (2002). 1-Cys peroxiredoxin overexpression protects cells against phospholipid peroxidation-mediated membrane damage. Proc. Natl. Acad. Sci. USA.

[49] Fisher A.B. (2017). Peroxiredoxin 6 in the repair of peroxidized cell membranes and cell signaling. Arch Biochem Biophys.

[50] Fisher A.B., Dodia C., Sorokina E.M., Li H., Zhou S., Raabe T., Feinstein S.I. (2016). A novel lysophosphatidylcholine acyl transferase activity is expressed by peroxiredoxin 6. J. Lipid Res..

[51] Manevich Y., Hutchens S., Halushka P., Tew K., Townsend D.M., Jauch E., Borg K. (2014). Peroxiredoxin VI oxidation in cerebrospinal fluid correlates with traumatic brain injury outcome. Free. Radic. Boil. Med..

[52] Chhunchha B., Kubo E., Singh P., Singh D.P. (2018). Sumoylation-deficient Prdx6 repairs aberrant Sumoylation-mediated Sp1 dysregulation-dependent Prdx6 repression and cell injury in aging and oxidative stress. Aging.

[53] Rhee S.G., Woo H.A., Kil I.S., Bae S.H. (2012). Peroxiredoxin functions as a peroxidase and a regulator and sensor of local peroxides. J. Biol. Chem..

[54] Wood Z.A., Schröder E., Harris J.R., Poole L.B. (2003). Structure, mechanism and regulation of peroxiredoxins. Trends Biochem. Sci..

[55] Fisher A.B. (2018). The phospholipase A2activity of peroxiredoxin 6. J. Lipid Res..

[56] Fisher A.B. (2011). Peroxiredoxin 6: A Bifunctional Enzyme with Glutathione Peroxidase and Phospholipase A2 Activities. Antioxidants Redox Signal..

[57] Chhunchha B., Fatma N., Kubo E., Singh D.P. (2014). Aberrant sumoylation signaling evoked by reactive oxygen species impairs protective function of Prdx6 by destabilization and repression of its transcription. FEBS J..

[58] Chhunchha B., Singh P., Singh D.P., Kubo E. (2018). Ginkgolic Acid Rescues Lens Epithelial Cells from Injury Caused by Redox Regulated-Aberrant Sumoylation Signaling by Reviving Prdx6 and Sp1 Expression and Activities. Int. J. Mol. Sci..

[59] Pak J.H., Choi H.-J., Choi C.Y., Tchah H. (2006). Expression of 1-Cys Peroxiredoxin in the Corneal Wound-Healing Process. Cornea.

[60] Pak J.H., Kim T.-I., Kim M.J., Kim J.Y., Choi H.-J., Kim S.A., Tchah H. (2006). Reduced expression of 1-cys peroxiredoxin in oxidative stress-induced cataracts. Exp. Eye Res..

[61] Munz B., Frank S., Hübner G., Olsen E., Werner S. (1997). A novel type of glutathione peroxidase: Expression and regulation during wound repair. Biochem. J..

[62] Wang X., Phelan S.A., Petros C., Taylor E.F., Ledinski G., Jürgens G., Forsman-Semb K., Paigen B. (2004). Peroxiredoxin 6 deficiency and atherosclerosis susceptibility in mice: Significance of genetic background for assessing atherosclerosis. Atherosclerosis.

[63] Houghton C.A., Fassett R.G., Coombes J.S. (2016). Sulforaphane and Other Nutrigenomic Nrf2 Activators: Can the Clinician′s Expectation Be Matched by the Reality?. Oxidative Med. Cell. Longev..

[64] Zanichelli F., Capasso S., Di Bernardo G., Cipollaro M., Pagnotta E., Cartenì M., Casale F., Iori R., Giordano A., Galderisi U. (2012). Low concentrations of isothiocyanates protect mesenchymal stem cells from oxidative injuries, while high concentrations exacerbate DNA damage. Apoptosis.

[65] Zanichelli F., Capasso S., Cipollaro M., Pagnotta E., Cartenì M., Casale F., Iori R., Galderisi U. (2012). Dose-dependent effects of R-sulforaphane isothiocyanate on the biology of human mesenchymal stem cells, at dietary amounts, it promotes cell proliferation and reduces senescence and apoptosis, while at anti-cancer drug doses, it has a cytotoxic effect. Age.

[66] Keum Y.-S. (2012). Regulation of Nrf2-Mediated Phase II Detoxification and Anti-oxidant Genes. Biomol. Ther..

[67] De Oliveira J.M.P.F., Remédios C., Oliveira H., Pinto P., Pinho F., Pinho S., Costa M., Santos C., De Oliveira J.M.F. (2014). Sulforaphane Induces DNA Damage and Mitotic Abnormalities in Human Osteosarcoma MG-63 Cells: Correlation with Cell Cycle Arrest and Apoptosis. Nutr. Cancer.

[68] Hanlon N., Coldham N., Gielbert A., Kuhnert N., Sauer M.J., King L.J., Ioannides C. (2008). Absolute bioavailability and dose-dependent pharmacokinetic behaviour of dietary doses of the chemopreventive isothiocyanate sulforaphane in rat. Br. J. Nutr..

[69] Riedl M.A., Saxon A., Diaz-Sanchez D. (2009). Oral sulforaphane increases Phase II antioxidant enzymes in the human upper airway. Clin. Immunol..

[70] Zhang D.D., Hannink M. (2003). Distinct Cysteine Residues in Keap1 Are Required for Keap1-Dependent Ubiquitination of Nrf2 and for Stabilization of Nrf2 by Chemopreventive Agents and Oxidative Stress. Mol. Cell. Boil..

[71] Ibaraki N., Chen S.C., Lin L.R., Okamoto H., Pipas J.M., Reddy V.N. (1998). Human lens epithelial cell line. Exp. Eye Res..

[72] Chhunchha B., Kubo E., Fatma N., Singh D.P. (2017). Sumoylation-deficient Prdx6 gains protective function by amplifying enzymatic activity and stability and escapes oxidative stress-induced aberrant Sumoylation. Cell Death Dis..

[73] Fatma N., Singh P., Chhunchha B., Kubo E., Shinohara T., Bhargavan B., Singh D.P. (2011). Deficiency of Prdx6 in lens epithelial cells induces ER stress response-mediated impaired homeostasis and apoptosis. Am. J. Physiol. Physiol..

[74] Chhunchha B., Singh P., Stamer W.D., Singh D.P. (2017). Prdx6 retards senescence and restores trabecular meshwork cell health by regulating reactive oxygen species. Cell Death Discov..

[75] Cory A.H., Owen T.C., Barltrop J.A., Cory J.G. (1991). Use of an Aqueous Soluble Tetrazolium/Formazan Assay for Cell Growth Assays in Culture. Cancer Commun..

[76] Fatma N., Kubo E., Sharma P., Beier D.R., Singh D.P. (2005). Impaired homeostasis and phenotypic abnormalities in Prdx6-/-mice lens epithelial cells by reactive oxygen species: Increased expression and activation of TGFbeta. Cell Death Differ..

[77] Fatma N., Singh D.P., Shinohara T., Chylack L.T. (2001). Transcriptional Regulation of the Antioxidant Protein 2 Gene, a Thiol-specific Antioxidant, by Lens Epithelium-derived Growth Factor to Protect Cells from Oxidative Stress. J. Boil. Chem..

[78] Kubo E., Hasanova N., Tanaka Y., Fatma N., Takamura Y., Singh D.P., Akagi Y. (2010). Protein expression profiling of lens epithelial cells from Prdx6-depleted mice and their vulnerability to UV radiation exposure. Am. J. Physiol. Cell Physiol..

[79] Kubo E., Singh D.P., Fatma N., Akagi Y. (2009). TAT-mediated peroxiredoxin 5 and 6 protein transduction protects against high-glucose-induced cytotoxicity in retinal pericytes. Life Sci..

[80] Singh D.P., Kubo E., Takamura Y., Shinohara T., Kumar A., Chylack L.T., Fatma N. (2006). DNA Binding Domains and Nuclear Localization Signal of LEDGF: Contribution of two Helix-Turn-Helix (HTH)-like Domains and a Stretch of 58 Amino Acids of the N-terminal to the Trans-activation Potential of LEDGF. J. Mol. Boil..

[81] Higgins L.G., Kelleher M.O., Eggleston I.M., Itoh K., Yamamoto M., Hayes J.D. (2009). Transcription factor Nrf2 mediates an adaptive response to sulforaphane that protects fibroblasts in vitro against the cytotoxic effects of electrophiles, peroxides and redox-cycling agents. Toxicol. Appl. Pharmacol..

[82] Chowdhury I., Mo Y., Gao L., Kazi A., Fisher A.B., Feinstein S.I. (2009). Oxidant stress stimulates expression of the human peroxiredoxin 6 gene by a transcriptional mechanism involving an antioxidant response element. Free Radic. Biol. Med..

[83] Lien Y.-C., Feinstein S.I., Dodia C., Fisher A.B. (2012). The Roles of Peroxidase and Phospholipase A2 Activities of Peroxiredoxin 6 in Protecting Pulmonary Microvascular Endothelial Cells Against Peroxidative Stress. Antioxidants Redox Signal..

[84] Harvey C.J., Thimmulappa R.K., Sethi S., Kong X., Yarmus L., Brown R.H., Feller-Kopman D., Wise R., Biswal S., David F.-K. (2011). Targeting Nrf2 signaling improves bacterial clearance by alveolar macrophages in patients with COPD and in a mouse model. Sci. Transl. Med..

[85] Bai Y., Cui W., Xin Y., Miao X., Barati M.T., Zhang C., Chen Q., Tan Y., Cui T., Zheng Y. (2013). Prevention by sulforaphane of diabetic cardiomyopathy is associated with up-regulation of Nrf2 expression and transcription activation. J. Mol. Cell. Cardiol..

[86] Cui W., Bai Y., Miao X., Luo P., Chen Q., Tan Y., Rane M.J., Miao L., Cai L. (2012). Prevention of Diabetic Nephropathy by Sulforaphane: Possible Role of Nrf2 Upregulation and Activation. Oxidative Med. Cell. Longev..

[87] Li B., Cui W., Liu J., Li R., Liu Q., Xie X.-H., Ge X.-L., Zhang J., Song X.-J., Wang Y. (2013). Sulforaphane ameliorates the development of experimental autoimmune encephalomyelitis by antagonizing oxidative stress and Th17-related inflammation in mice. Exp. Neurol..

[88] Miao X., Bai Y., Sun W., Cui W., Xin Y., Wang Y., Tan Y., Miao L., Fu Y., Su G. (2012). Sulforaphane prevention of diabetes-induced aortic damage was associated with the up-regulation of Nrf2 and its down-stream antioxidants. Nutr. Metab..

[89] Shapiro T.A., Fahey J.W., Dinkova-Kostova A.T., Holtzclaw W.D., Stephenson K.K., Wade K.L., Ye L., Talalay P. (2006). Safety, Tolerance, and Metabolism of Broccoli Sprout Glucosinolates and Isothiocyanates: A Clinical Phase I Study. Nutr. Cancer.

[90] He X.-Y., Zhao G.-J., Lu Z.-Q., Hong G.-L., He F., Liang H., Qiu Q.-M., Li J.-R. (2009). Oxidative stress of acute paraquat poisoned rats and sodium dimercaptopropane sulfonate intervention. Chin. J. Ind. Hyg. Occup. Dis..

[91] He X., Ma Q. (2009). NRF2 cysteine residues are critical for oxidant/electrophile-sensing, Kelch-like ECH-associated protein-1-dependent ubiquitination-proteasomal degradation, and transcription activation. Mol. Pharmacol..

[92] He X., Kan H., Cai L., Ma Q. (2009). Nrf2 is critical in defense against high glucose-induced oxidative damage in cardiomyocytes. J. Mol. Cell. Cardiol..

[93] Zheng H., Whitman S.A., Wu W., Wondrak G.T., Wong P.K., Fang D., Zhang D.D. (2011). Therapeutic Potential of Nrf2 Activators in Streptozotocin-Induced Diabetic Nephropathy. Diabetes.

[94] Kim J.Y., Park H.J., Um S.H., Sohn E.H., Kim B.O., Moon E.Y., Rhee D.K., Pyo S. (2012). Sulforaphane suppresses vascular adhesion molecule-1 expression in TNF-alpha-stimulated mouse vascular smooth muscle cells: Involvement of the MAPK, NF-kappaB and AP-1 signaling pathways. Vasc. Pharmacol..

[95] Kim J.K., Park S.U. (2016). Current potential health benefits of sulforaphane. EXCLI J..

[96] Kim J.H., Ki H.K., Jung J.Y., Han H.S., Shim J.H., Oh S., Choi K.H., Choi E.S., Shin J.A., Leem D.H. (2010). Sulforaphane Increases Cyclin-Dependent Kinase Inhibitor, p21 Protein in Human Oral Carcinoma Cells and Nude Mouse Animal Model to Induce G(2)/M Cell Cycle Arrest. J. Clin. Biochem. Nutr..

[97] Xu Z., Wang S., Ji H., Zhang Z., Chen J., Tan Y., Wintergerst K., Zheng Y., Sun J., Cai L. (2016). Broccoli sprout extract prevents diabetic cardiomyopathy via Nrf2 activation in db/db T2DM mice. Sci. Rep..

[98] Singh S.V., Srivastava S.K., Choi S., Lew K.L., Antosiewicz J., Xiao D., Zeng Y., Watkins S.C., Johnson C.S., Trump D.L. (2005). Sulforaphane-induced Cell Death in Human Prostate Cancer Cells Is Initiated by Reactive Oxygen Species. J. Boil. Chem..

[99] Suzuki T., Yamamoto M. (2015). Molecular basis of the Keap1–Nrf2 system. Free. Radic. Boil. Med..

[100] Morimitsu Y., Nakagawa Y., Hayashi K., Fujii H., Kumagai T., Nakamura Y., Osawa T., Horio F., Itoh K., Iida K. (2002). A sulforaphane analogue that potently activates the Nrf2-dependent detoxification pathway. J. Biol. Chem..

[101] Sun Z., Huang Z., Zhang D.D. (2009). Phosphorylation of Nrf2 at Multiple Sites by MAP Kinases Has a Limited Contribution in Modulating the Nrf2-Dependent Antioxidant Response. PLoS ONE.

[102] Finkel T., Holbrook N.J. (2000). Oxidants, oxidative stress and the biology of ageing. Nature.

[103] Jaramillo M.C., Zhang D.D. (2013). The emerging role of the Nrf2–Keap1 signaling pathway in cancer. Genes Dev..

[104] DeNicola G.M., Karreth F.A., Humpton T.J., Gopinathan A., Wei C., Frese K., Mangal D., Yu K.H., Yeo C.J., Calhoun E.S. (2011). Oncogene-induced Nrf2 transcription promotes ROS detoxification and tumorigenesis. Nature.

[105] Han Z., Xu Q., Li C., Zhao H. (2017). Effects of sulforaphane on neural stem cell proliferation and differentiation. Genesis.

[106] Zhao H.-D., Zhang F., Shen G., Li Y.-B., Li Y.-H., Jing H.-R., Ma L.-F., Yao J.-H., Tian X.-F. (2010). Sulforaphane protects liver injury induced by intestinal ischemia reperfusion through Nrf2-ARE pathway. World J. Gastroenterol..

[107] Keum Y.-S. (2011). Regulation of the Keap1/Nrf2 system by chemopreventive sulforaphane: Implications of posttranslational modifications. Ann. New York Acad. Sci..

[108] Keum Y.-S., Yu S., Chang P.P.-J., Yuan X., Kim J.-H., Xu C., Han J., Agarwal A., Kong A.-N.T. (2006). Mechanism of Action of Sulforaphane: Inhibition of p38 Mitogen-Activated Protein Kinase Isoforms Contributing to the Induction of Antioxidant Response Element–Mediated Heme Oxygenase-1 in Human Hepatoma HepG2 Cells. Cancer Res..

[109] Magesh S., Chen Y., Hu L. (2012). Small Molecule Modulators of Keap1-Nrf2-ARE Pathway as Potential Preventive and Therapeutic Agents. Med. Res. Rev..

[110] Manevich Y., Shuvaeva T., Dodia C., Kazi A., Feinstein S.I., Fisher A.B. (2009). Binding of peroxiredoxin 6 to substrate determines differential phospholipid hydroperoxide peroxidase and phospholipase A2 activities. Arch. Biochem. Biophys..

[111] Liu H., Smith A.J.O., Lott M.C., Bao Y., Bowater R.P., Reddan J.R., Wormstone M. (2013). Sulforaphane Can Protect Lens Cells Against Oxidative Stress: Implications for Cataract Prevention. Investig. Opthalmology Vis. Sci..

[112] Eren E., Tufekci K.U., Isci K.B., Tastan B., Genc K., Genc S. (2018). Sulforaphane Inhibits Lipopolysaccharide-Induced Inflammation, Cytotoxicity, Oxidative Stress, and miR-155 Expression and Switches to Mox Phenotype through Activating Extracellular Signal-Regulated Kinase 1/2–Nuclear Factor Erythroid 2-Related Factor 2/Antioxidant Response Element Pathway in Murine Microglial Cells. Front. Immunol..

[113] Gu J., Cheng Y., Wu H., Kong L., Wang S., Xu Z., Zhang Z., Tan Y., Keller B.B., Zhou H. (2017). Metallothionein Is Downstream of Nrf2 and Partially Mediates Sulforaphane Prevention of Diabetic Cardiomyopathy. Diabetes.

[114] Rocourt C.R., Wu M., Chen B.P., Cheng W.H. (2013). The catalytic subunit of DNA-dependent protein kinase is downstream of A™ and feeds forward oxidative stress in the selenium-induced senescence response. J. Nutr. Biochem..

[115] Biton S., Ashkenazi A. (2011). NEMO and RIP1 control cell fate in response to extensive DNA damage via TNF-alpha feedforward signaling. Cell.

[116] Kubo E., Fatma N., Akagi Y., Beier D.R., Singh S.P., Singh D.P. (2008). TAT-mediated PRDX6 protein transduction protects against eye lens epithelial cell death and delays lens opacity. Am. J. Physiol. Physiol..

[117] Kovac S., Angelova P.R., Holmstrom K.M., Zhang Y., Dinkova-Kostova A.T., Abramov A.Y. (2015). Nrf2 regulates ROS production by mitochondria and NADPH oxidase. Biochim. Biophys. Acta.

[118] Bleier L., Wittig I., Heide H., Steger M., Brandt U., Dröse S. (2015). Generator-specific targets of mitochondrial reactive oxygen species. Free. Radic. Boil. Med..

[119] Adimora N.J., Jones D.P., Kemp M.L. (2010). A Model of Redox Kinetics Implicates the Thiol Proteome in Cellular Hydrogen Peroxide Responses. Antioxidants Redox Signal..

[120] Quinlan C.L., Goncalves R.L.S., Hey-Mogensen M., Yadava N., Bunik V.I., Brand M.D. (2014). The 2-Oxoacid Dehydrogenase Complexes in Mitochondria Can Produce Superoxide/Hydrogen Peroxide at Much Higher Rates Than Complex, I.J. Boil. Chem..

[121] Campbell M.R., Karaca M., Adamski K.N., Chorley B.N., Wang X., Bell D.A. (2013). Novel Hematopoietic Target Genes in the NRF2-Mediated Transcriptional Pathway. Oxidative Med. Cell. Longev..

[122] Chorley B.N., Campbell M.R., Wang X., Karaca M., Sambandan D., Bangura F., Xue P., Pi J., Kleeberger S.R., Bell D.A. (2012). Identification of novel NRF2-regulated genes by ChIP-Seq: Influence on retinoid X receptor alpha. Nucleic Acids Res..

[123] Subedi L., Lee J.H., Yumnam S., Ji E., Kim S.Y. (2019). Anti-Inflammatory Effect of Sulforaphane on LPS-Activated Microglia Potentially through JNK/AP-1/NF-kappaB Inhibition and Nrf2/HO-1 Activation. Cells.

[124] Nair S., Barve A., Khor T.-O., Shen G.-X., Lin W., Chan J.Y., Cai L., Kong A.-N. (2010). Regulation of Nrf2- and AP-1-mediated gene expression by epigallocatechin-3-gallate and sulforaphane in prostate of Nrf2-knockout or C57BL/6J mice and PC-3 AP-1 human prostate cancer cells. Acta Pharmacol. Sin..

[125] Beaver L.M., Buchanan A., Sokolowski E.I., Riscoe A.N., Wong C.P., Chang J.H., Löhr C.V., Williams D.E., Dashwood R.H., Ho E. (2014). Transcriptome analysis reveals a dynamic and differential transcriptional response to sulforaphane in normal and prostate cancer cells and suggests a role for Sp1 in chemoprevention. Mol. Nutr. Food Res..

[126] Chew Y.C., Adhikary G., Wilson G.M., Xu W., Eckert R.L. (2012). Sulforaphane Induction of p21Cip1 Cyclin-dependent Kinase Inhibitor Expression Requires p53 and Sp1 Transcription Factors and Is p53-dependent*. J. Boil. Chem..

[127] Liu Y., Liu P., Wang Q., Sun F., Liu F. (2019). Sulforaphane Attenuates H(2)O(2)-induced Oxidant Stress in Human Trabecular Meshwork Cells (H™Cs) via the Phosphatidylinositol 3-Kinase (PI3K)/Serine/Threonine Kinase (Akt)-Mediated Factor-E2-Related Factor 2 (Nrf2) Signaling Activation. Med. Sci. Monit..

